# Effect of Comprehensive Care Coordination on Medicaid Expenditures Compared With Usual Care Among Children and Youth With Chronic Disease: A Randomized Clinical Trial

**DOI:** 10.1001/jamanetworkopen.2019.12604

**Published:** 2019-10-04

**Authors:** Rachel Caskey, Kellyn Moran, Daniel Touchette, Molly Martin, Garret Munoz, Pinal Kanabar, Benjamin Van Voorhees

**Affiliations:** 1 Department of Medicine, University of Illinois at Chicago; 2 Department of Pediatrics, University of Illinois at Chicago; 3 College of Pharmacy, University of Illinois at Chicago; 4 Research Resource Center, University of Illinois at Chicago

## Abstract

**Importance:**

Medicaid spending on children and young adults with chronic disease could be decreased through care coordination programs by reducing unnecessary hospital and emergency care.

**Objective:**

To assess whether a comprehensive care coordination program reduces Medicaid expenditures by decreasing hospital and emergency department (ED) utilization.

**Design, Setting, and Participants:**

This randomized clinical trial included 6259 children and young adults with chronic disease who received public insurance through Illinois Medicaid. In April 2016, eligible youth were randomized to receive comprehensive care coordination through the Coordinated Healthcare for Complex Kids (CHECK) program (n = 3126) or usual care (n = 3119) to measure the effect of the CHECK program on Medicaid expenditures and health care utilization using a difference-in-differences (DID) approach. Data were collected from May 1, 2014, to April 30, 2017, and analyzed in May 2018.

**Interventions:**

Care coordination, mental health care, education, and social support were provided to CHECK participants and their family members. Services were tailored based on family and participant need.

**Main Outcomes and Measures:**

Mean annual Medicaid expenditures, mean annual health care utilization by category (ED and inpatient), and chronic disease type and risk level.

**Results:**

A total of 6259 participants (mean [SD] age, 11.3 [6.4] years; 2918 [46.6%] female; 2594 [41.4%] with medium and high risk) were randomized. Following the exclusion of 14 outliers, 6245 participants were analyzed. The mean (SD) annual Medicaid expenditure before the intervention was $1633 ($4006) for the intervention group and $1703 ($4466) for the usual care group, which decreased to a mean (SD) of $1341 ($3004) and $1413 ($3785), respectively, after the intervention (DID, −$1; 95% CI, −$199 to $196; *P* = .99). The mean (SD) inpatient utilization before the intervention was 63.0 (344.4) per 1000 person-years (PYs) for the intervention group and 69.3 (370.9) per 1000 PYs for the usual care group, which decreased to 43.5 (297.2) per 1000 PYs and 47.8 (304.9) per 1000 PYs, respectively, after the intervention (DID, 2.0; 95% CI, −17.9 to 21.8; *P* = .85). Among participants with asthma, those in the intervention group had a greater mean (SD) decrease in ED utilization compared with usual care, but the difference was not significant (−225.9 [65.3] vs −104.5 [80.0] visits per 1000 PY; DID, −121.5; 95% CI, −268.9 to 26.0; *P* = .11). Similarly, enrolled participants with sickle cell disease had a smaller but not significant mean (SD) increase in ED utilization compared with usual care (583.3 [839.0] vs 3761.9 [4611.2] visits per 1000 PYs; DID, −3178.6; 95% CI, −10 724.3 to 4367.2; *P* = .41).

**Conclusions and Relevance:**

Overall Medicaid expenditures and health care utilization (hospital and ED) decreased similarly for both CHECK participants and the usual care group.

**Trial Registration:**

ClinicalTrials.gov identifier: NCT04057521

## Key Points

**Question:**

Does a comprehensive care coordination program for publicly insured children and young adults with chronic disease decrease Medicaid expenditures by decreasing hospital and emergency department utilization?

**Findings:**

In this randomized clinical trial, 3126 participants were randomized to care coordination and 3119 participants were randomized to usual care. Medicaid expenditures and health care utilization decreased similarly for both groups during the first year of the care coordination program.

**Meaning:**

Care coordination did not reduce Medicaid expenditures or hospital and emergency department utilization among children and young adults with chronic disease.

## Teaser

This randomized clinical trial assesses whether a comprehensive care coordination program reduces Medicaid expenditures by decreasing hospital and emergency department utilization among publicly insured children and youth.

## Introduction

The number of children with chronic disease is increasing in the United States, with nearly 25% of children having been diagnosed with at least 1 chronic medical condition.^1,2^ Minority and low-income children are more likely to have a chronic disease and poorer health outcomes compared with non-Hispanic white children and children with higher income.^1^ Children with chronic medical conditions consume a disproportionate share of the pediatric health care expenditures in the United States, especially for inpatient care.^3^ Small-scale studies have demonstrated that an enhanced medical home model can decrease health care costs among children with chronic disease.^4^ Similarly, coordination of care has shown promise in improving care delivery and outcomes among children with asthma.^5^ However, a hospital-based comprehensive case management program resulted in an increase in cost among medically complex children.^6^ Further, studies of large-scale care coordination programs for Medicaid and Medicare beneficiaries have demonstrated variable impact on expenditures, with some programs decreasing cost while others appear to have had no impact.^7,8,9^

In 2014, the University of Illinois Health and Health Sciences System developed a comprehensive care coordination demonstration project designed to provide services for children and young adults with chronic health conditions living in Chicago, Illinois, who are insured by Medicaid. This program, Coordinated Healthcare for Complex Kids (CHECK), was funded by a Centers for Medicare & Medicaid Services Innovation (CMMI) Award.^10^ The CHECK model took a broad approach to care coordination and health promotion by addressing social determinants of health, caregiver wellness, and mental health needs in addition to chronic disease management.^11^ The program targeted children and young adults with diagnoses of asthma, diabetes, sickle cell disease, seizure disorder, or prematurity from birth to age 25 years. All participants were enrolled in the traditional, fee-for-service state Medicaid program or a Medicaid managed care organization (MCO) in Illinois. The CHECK program provided access to multiple services, including care coordination delivered by community health workers (CHWs), mental health services delivered by mental health professionals, and disease-specific health education. Community health workers assessed individual and family needs as well as patterns of health care utilization during the year prior to enrollment to determine specific services offered to each family. For example, the family of a child with uncontrolled asthma and multiple emergency department (ED) visits may have received a home visit by a CHW to evaluate potential environmental triggers, review medications, and provide in-depth asthma education, followed by monthly telephone calls to check in on the child and family.

The primary aim of the CHECK program was to decrease Medicaid expenditures during a 3-year period by decreasing unnecessary ED visits and hospitalizations. We had a unique opportunity to prospectively randomize participants to receive CHECK vs usual care (UC) to evaluate the program; this provided analytic rigor not available to many large-scale care delivery programs. This report describes an analysis evaluating the effect of the CHECK program on participants’ Medicaid expenditures and ED and inpatient utilization.

## Methods

The objective of this study was to compare the effect of the CHECK program on Medicaid expenditures among a subgroup of eligible CHECK participants who were randomized to either the CHECK program or UC. The trial protocol is available in Supplement 1. The study was approved by the University of Illinois at Chicago institutional review board. This trial was not registered prospectively. The project started as an observational cohort study. The methodology was changed to a randomized clinical trial when we had the opportunity to randomize a large group of patients to receive the intervention or UC. This was done as part of the independent evaluation of the CMMI program. We did not understand the requirement to register this trial before it was conducted. At the request of the editors, we registered the trial retrospectively on August 15, 2019. This study follows the Consolidated Standards of Reporting Trials (CONSORT) reporting guideline.

### Study Design and Sample

Eligible participants were identified for enrollment using Medicaid claims data and were passively enrolled over 2 years (2014-2016). Eligibility criteria were as follows: (1) age 0 to 25 years; (2) documentation of asthma, diabetes (type 1 or 2), prematurity, seizure disorder, or sickle cell disease; (3) enrolled in Medicaid; and (4) resided in Cook County, Illinois. Beneficiaries of either the traditional state Medicaid program or a Medicaid MCO were included. *International Classification of Diseases, Ninth Revision, Clinical Modification *(*ICD*-*9*-*CM*) and *ICD*-*10*-*CM* codes were used to determine presence of eligible chronic diseases (eTable 1 in Supplement 2). Participants with more than 1 CHECK diagnosis were categorized in each category (eg, a child with sickle cell disease and asthma would be categorized in both categories). Enrolled participants who did not have a targeted chronic disease were retained in the program and designated as having no CHECK diagnosis for the purpose of analysis.

Eligible participants were considered enrolled in CHECK after being provided a letter of notification of passive enrollment into the program. Enrolled participants were risk stratified using claims data based on health care utilization during the 12 months prior to enrollment as follows: (1) low risk if no hospitalizations or ED visits, (2) medium risk if 1 hospitalization and/or 1 to 3 ED visits, and (3) high risk if more than 1 hospitalization and/or more than 3 ED visits. Once an enrolled participant was contacted by the CHECK program and completed an initial health assessment, a care plan was created and the participant was considered engaged*.* By design, not all enrolled participants were contacted for engagement. Initial engagement efforts focused on all participants with medium and high risk, followed by participants with low risk. Those who could not be contacted or did not complete the engagement process for any reason remained categorized as enrolled.

### Randomization

Though CHECK was initially a care delivery demonstration project, in 2016 we had the opportunity to randomize 6259 participants who met eligibility criteria for CHECK to evaluate the program. These participants had no previous exposure to CHECK and were randomized to either enrollment in the CHECK program or UC. Purposive (by age, type of chronic disease, and risk level) nonblinded randomization was conducted in April 2016 by a national research group that performs external auditing for the CMMI awards (Figure). Participants randomized to CHECK received a letter notifying the family of enrollment in the program, per usual CHECK protocol. Informed consent was waived because this was a population-based demonstration program and no individual-level clinical data were collected.

**Figure.**
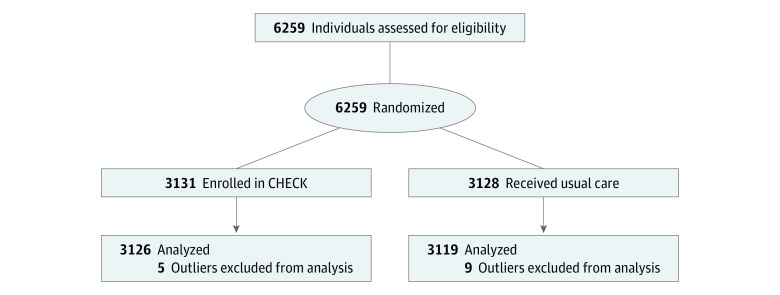
Flow Diagram of Study Outlier refers to participants who had health care expenditures of more than $100 000 of paid claims in the years before and after randomization. CHECK indicates Coordinated Healthcare for Complex Kids.

### Data

We analyzed Illinois Medicaid paid claims for CHECK participants using the Care Coordination Claims Data (CCCD) provided by the Illinois Department of Healthcare and Family Services. Information from CCCD was provided to CHECK on a monthly basis for all enrolled participants. This data set comprised all claims paid by Medicaid, including prescription drugs, for the period from May 1, 2014, to April 30, 2017. Analysis was conducted in May 2018 (12 months after April 2017) to ensure that more than 95% of claims had been processed and adjudicated prior to analysis. To normalize expenditures, we used the CCCD data for participants enrolled in a Medicaid MCO (what the state Medicaid program would pay for the same claim) to calculate expenditures. The data set includes participant’s age, sex, address, diagnoses (from billing diagnostic codes), procedure codes, dates of service, and payment amount per claim.

### Outcomes

Baseline demographic and clinical variables were identified from the CCCD, including age, sex, diagnoses, baseline CHECK risk tier, and Chronic Illness and Disability Payment System risk score.^12,13^ This score is a claims-based score commonly used by payers to risk stratify beneficiaries and was included to allow comparison of participants in CHECK with other cohorts.^13^

The number of inpatient hospitalizations per patient was identified using inpatient facility claims. Claims for the same patient identifier with overlapping service dates were considered a single hospitalization. The number of ED visits per patient was identified from outpatient facility claims or professional claims with revenue codes indicating an ED place of service or *Current Procedural Terminology* codes for emergency services. Outpatient visits were defined as outpatient facility claims or professional service claims with evaluation and management codes for office visits (excluding observation or outpatient surgery). Owing to the fact that professional service claims may be submitted independently from facility claims, claims with the same patient identification number, national provider identification, and service date were considered the same event to avoid duplication.

Expenditures were measured for each patient overall and by category of service (ie, inpatient visit, ED visit, outpatient visit, prescriptions, and other). Prescription expenditures were obtained from the CCCD prescription drug file. For inpatient, ED, and outpatient visit expenditures, costs associated with any remaining outpatient or professional claims with overlapping dates of service were considered part of the event. Total expenditures per patient were defined as the sum of all paid claims for any covered service. Expenditures not attributable to inpatient, ED, outpatient visits, or prescription costs were considered other and included services such as observation, outpatient surgery, dental care, mental health, laboratory tests, and radiology.

### Statistical Analysis

A randomized clinical trial design was used to compare the effect of the CHECK program on expenditures and utilization during the first year of the program with expenditures and utilization in the year prior to randomization and with participants in the UC group. Outcomes were measured for each study group (CHECK and UC) between May 1, 2015, and April 30, 2016, for prerandomization and between May 1, 2016, and April 30, 2017, for postrandomization.

Demographic and clinical characteristics were described using means and SDs for normally distributed continuous variables, medians and interquartile ranges for nonnormally distributed continuous variables, and frequencies and percentages for categorical variables. Bivariate comparisons were made for all variables between the 2 study groups within each cohort using 2-sample *t* tests, the Wilcoxon rank sum test, or χ^2^ test as appropriate to variable type.

The effect of the CHECK program on the change in expenditures and resource utilization was evaluated using a difference-in-differences (DID) method. This method compared outcomes occurring in the 12 months prior to the randomization date with outcomes occurring in the 12 months after the randomization date and assessed the difference in change occurring across time between groups. To assess the assumption of parallel trends, we partitioned the prerandomization data into 6-month increments. We observed no statistically significant difference in the trend of total cost between the groups before the randomization date; thus, the assumption of parallel trends was met (eFigure in Supplement 2). This approach addressed potential baseline differences between the 2 groups that may have occurred despite randomization.

The difference in the change in mean expenditures (total and by expenditure type) was estimated using a generalized estimating equation with a γ distribution, log link and indicator variables for group, period, and an interaction of group and period. The coefficient for the interaction term produced by this model is the DID estimate. Differences in the change in the rate of inpatient, ED, and outpatient visits per 1000 person-years (PYs) were estimated using a generalized estimating equation model with negative binomial distribution to account for overdispersion.

Participants with serious medical conditions (eg, genetic disorders or congenital defects) resulting in health care expenditures of more than $100 000 of paid claims in the years before and after randomization were excluded from analyses as outliers. Based on their unique medical needs, these participants were determined not representative of a typical population of children and young adults with chronic disease.

All data were analyzed using SAS software version 9.4 (SAS Institute Inc). Statistical significance was set at *P* < .05, and all tests were 2-tailed.

## Results

Our cohort included 6259 participants (mean [SD] age, 11.3 [6.4] years; 2918 [46.6%] female; 2594 [41.4%] with medium and high risk). We excluded 14 outliers, resulting in an analytic group of 6245 randomized participants (3126 [50.1%] in the intervention group; 3119 [49.9%] in the UC group) (Figure). No participants were lost to follow-up, and all analysis was by assigned group. All participants had continuous enrollment in Medicaid during the analytic period. The intervention and UC groups were similar; the majority of participants had asthma (2098 [67.1%] vs 2068 [66.9%]), and a similar number had no CHECK diagnosis (762 [24.4%] vs 769 [24.7%]). The majority were classified as low risk (1840 [58.9%] vs 1823 [58.5%]), and a similar number were classified as medium risk (1057 [33.8%] vs 1078 [34.6%]). Approximately 60% were 12 years or younger in both groups (1844 [59.0%] vs 1834 [58.8%]) (Table 1).

**Table 1.** Participant Characteristics

Characteristic	No. (%)	
	CHECK Group (n = 3126)	UC Group (n = 3119)
Age at enrollment, y		
Mean (SD)	11.3 (6.4)	11.4 (6.4)
Median (IQR)	11.0 (6.0-16.0)	11.0 (6.0-16.0)
No. (%)		
≥18	596 (19.1)	598 (19.2)
13-17	686 (21.9)	687 (22.0)
6-12	1163 (37.2)	1161 (37.2)
1-5	660 (21.1)	653 (20.9)
<1	21 (0.7)	20 (0.6)
Sex		
Male	1670 (53.4)	1663 (53.3)
Female	1456 (46.6)	1456 (46.7)
CHECK diagnosis		
Asthma	2098 (67.1)	2068 (66.9)
Diabetes	166 (5.3)	159 (5.1)
Prematurity	70 (2.2)	69 (2.2)
Seizure disorder	107 (3.4)	113 (3.6)
Sickle cell disease	12 (0.4)	21 (0.7)
None	762 (24.4)	769 (24.7)
Baseline risk tier		
Low	1840 (58.9)	1823 (58.5)
Medium	1057 (33.8)	1078 (34.6)
High	229 (7.3)	218 (7.0)
CDPS risk score		
Mean (SD)	0.5 (0.6)	0.5 (0.6)
Median (IQR)	0.2 (0.2-0.5)	0.2 (0.2-0.5)

Abbreviations: CDPS, Chronic Illness and Disability Payment System; CHECK, Coordinated Healthcare for Complex Kids; IQR, interquartile range; UC, usual care.

### Medicaid Expenditures

During the analytic period, total expenditures decreased for both groups (Table 2). Before the intervention, the mean (SD) total annual health care expenditure was $1633 ($4006) for the intervention group and $1703 ($4466) for the UC group, which decreased to a mean (SD) of $1341 ($3004) and $1413 ($3785), respectively, after the intervention (DID, −$1; 95% CI, −$199 to $196; *P* = .99). This decrease in Medicaid expenditures was similar by type of expenditure among both groups. Mean (SD) annual inpatient expenditures decreased from $347 ($2876) preintervention to $222 ($1710) postintervention among the intervention group and from $396 ($3196) preintervention to $285 ($2568) postintervention among the UC group (DID, −$13; 95% CI, −$173 to $147; *P* = .87). Similarly, mean (SD) annual ED expenditures decreased from $168 ($456) preintervention to $145 ($417) postintervention among the intervention group and from $169 ($411) preintervention to $152 ($482) postintervention among the UC group (DID, −$7; 95% CI, −$32 to $18; *P* = .59) (Table 2).

**Table 2.** Mean Annual Expenditures Before and After Intervention for 3126 Participants in the CHECK Group and 3119 Participants in the UC Group

Expenditure Category	Mean (SD) Expenditure, $						DID (95% CI), $	*P* Value
	12 mo Before Intervention		12 mo After Intervention		Difference			
	CHECK Group	UC Group	CHECK Group	UC Group	CHECK Group	UC Group		
Total	1633 (4006)	1703 (4466)	1341 (3004)	1413 (3785)	−292 (90)	−290 (105)	−1 (−199 to 196)	.99
Inpatient	347 (2876)	396 (3196)	222 (1710)	285 (2568)	−125 (60)	−111 (73)	−13 (−173 to 147)	.87
Emergency department	168 (456)	169 (411)	145 (417)	152 (482)	−23 (11)	−17 (11)	−7 (−32 to 18)	.59
Outpatient visits	235 (303)	234 (299)	196 (274)	189 (251)	−39 (7)	−45 (7)	6 (−8 to 19)	.42
Prescriptions	353 (1213)	388 (1659)	345 (1543)	357 (1736)	−8 (35)	−31 (43)	23 (−34 to 80)	.42
Other^a^	530 (1392)	516 (1158)	433 (980)	430 (1085)	−97 (30)	−86 (28)	−10 (−69 to 48)	.73

Abbreviations: CHECK, Coordinated Healthcare for Complex Kids; DID, difference-in-differences; UC, usual care.

^a^ Includes all remaining Medicaid paid claims (eg, dental care, mental health, ancillary services, laboratory tests, and radiology).

### Inpatient and ED Utilization

The rate of inpatient and ED utilization decreased for both groups. The mean (SD) inpatient utilization before enrollment in CHECK was 63.0 (344.4) per 1000 PYs for the intervention group and 69.3 (370.9) per 1000 PYs for the UC group, which decreased to 43.5 (297.2) per 1000 PYs and 47.8 (304.9) per 1000 PYs, respectively, after the intervention (DID, 2.0; 95% CI, −17.9 to 21.8; *P* = .85) (Table 3). Similarly, during the preintervention year, the mean (SD) ED utilization was 961.0 (2049.7) per 1000 PYs for the intervention group and 959.6 (2094.7) per 1000 PYs for the UC group, which decreased to 768.7 (1868.6) per 1000 PYs and 825.3 (2471.8) per 1000 PYs, respectively, postintervention (DID, −57.9; 95% CI, −166.2 to 50.4; *P* = .29) (Table 3).

**Table 3.** Inpatient and ED Utilization, Before and After Intervention, by Disease and Risk

Service Location	Mean (SD) No. of Visits/1000 PYs						DID (95% CI), No. of Visits/1000 PYs	*P* Value
	12 mo Before Intervention		12 mo After Intervention		Difference			
	CHECK Group	UC Group	CHECK Group	UC Group	CHECK Group	UC Group		
**Entire Cohort: 3126 Participants in CHECK Group and 3119 Participants in UC Group**								
Inpatient	63.0 (344.4)	69.3 (370.9)	43.5 (297.2)	47.8 (304.9)	−19.5 (−8.1)	−21.5 (−8.6)	2.0 (−17.9 to 21.8)	.85
ED	961.0 (2049.7)	959.6 (2094.7)	768.7 (1868.6)	825.3 (2471.8)	−192.3 (49.6)	−134.3 (58.0)	−57.9 (−166.2 to 50.4)	.29
**Asthma: 2098 Participants in CHECK Group and 2068 Participants in UC Group**								
Inpatient	58.2 (326.0)	68.5 (377.5)	50.0 (338.1)	52.2 (319.8)	−8.1 (10.3)	−16.3 (10.9)	8.2 (−16.1 to 32.4)	.51
ED	1083.9 (2206.3)	1070.9 (2243.3)	858.0 (2020.1)	966.5 (2863.1)	−225.9 (65.3)	−104.5 (80.0)	−121.5 (−268.9 to 26.0)	.11
**Diabetes: 166 Participants in CHECK Group and 159 Participants in UC Group**								
Inpatient	192.8 (737.9)	182.4 (692.2)	144.6 (430.2)	157.2 (413.9)	−48.2 (66.3)	−25.2 (64.0)	−23.0 (−164.8 to 118.7)	.75
ED	1397.6 (2660.3)	1566.0 (3870.6)	1234.9 (2162.8)	1257.9 (2501.2)	−162.7 (266.1)	−308.2 (365.5)	145.5 (−424.8 to 715.9)	.62
**Prematurity: 70 Participants in CHECK Group and 69 Participants in UC Group**								
Inpatient	357.1 (660.2)	231.9 (518.6)	57.1 (289.2)	0	−300.0 (86.1)	−231.9 (62.4)	−68.1 (not estimated)	NA
ED	1700.0 (2515.7)	1333.3 (2207.4)	1328.6 (2387.9)	884.1 (1649.7)	−371.4 (414.6)	−449.3 (331.8)	77.9 (−738.5 to 894.2)	.85
**Seizure Disorder: 107 Participants in CHECK Group and 113 Participants in UC Group**								
Inpatient	168.2 (504.4)	345.1 (821.3)	84.1 (310.9)	132.7 (559.1)	−84.1 (57.3)	−212.4 (93.5)	128.3 (−74.3 to 330.8)	.21
ED	1579.4 (2638.7)	2274.3 (3360.1)	1486.0 (3103.1)	1681.4 (3071.4)	−93.5 (393.8)	−592.9 (428.2)	499.5 (−231.1 to 1230.0)	.18
**Sickle Cell: 12 Participants in CHECK Group and 21 Participants in UC Group**								
Inpatient	500.0 (1000.0)	476.2 (1030.5)	333.3 (651.3)	47.6 (218.2)	−166.7 (344.5)	−428.6 (229.9)	261.9 (−590.3 to 1114.1)	.55
ED	1750.0 (2094.4)	2095.2 (3590.3)	2333.3 (2015.1)	5857.1 (20 823.8)	583.3 (839.0)	3761.9 (4611.2)	−3178.6 (−10 724.3 to 4367.2)	.41
**Participants With Medium and High Risk: 1286 Participants in CHECK Group and 1296 Participants in UC Group**								
Inpatient	153.2 (524.0)	166.7 (561.3)	77.0 (424.3)	85.6 (435.4)	−76.2 (18.8)	−81.0 (19.7)	4.8 (−40.8 to 50.4)	.84
ED	2335.9 (2646.3)	2309.4 (2728.5)	1329.7 (2645.3)	1459.9 (3617.6)	−1006.2 (104.3)	−849.2 (125.9)	−156.7 (−395.3 to 81.9)	.20

Abbreviations: CHECK, Coordinated Healthcare for Complex Kids; DID, difference-in-differences; ED emergency department; NA, not applicable; PY, person-year; UC, usual care.

The decrease in utilization was similar between the intervention and UC groups when stratified by disease type. Participants in the CHECK program with asthma had a larger mean (SD) decrease in ED utilization (−225.9 [65.3] visits per 1000 PYs) compared with participants in the UC group (−104.5 [80.0] per 1000 PYs), although the change was not statistically significant (DID, −121.5; 95% CI, −268.9 to 26.0; *P* = .11) (Table 3). Participants in the intervention group with sickle cell disease had a smaller mean (SD) increase in ED utilization compared with participants in the UC group (583.3 [839.0] vs 3761.9 [4611.2] visits per 1000 PYs), but this was not statistically significant (DID, −3178.6; 95% CI, −10 724.3 to 4367.2; *P* = .41) (Table 3). Analysis by risk group found that participants with medium and high risk had a similar mean (SD) decrease in ED utilization among the intervention group compared with the UC group (−1006.2 [104.3] vs −849.5 [125.9] per 1000 PYs; DID, −156.7; 95% CI, −395.3 to 81.9; *P* = .20) (Table 3).

To evaluate the impact of removing outliers, an analysis was conducted by repeating all expenditure and utilization analyses while including participants with more than $100 000 in annual expenditures (eTable 2 and eTable 3 in Supplement 2). Inclusion of outliers in the UC group (n = 9) resulted in a 40% increase in mean total expenditures in the year prior to randomization. Inclusion of the outliers in the CHECK group (n = 5) resulted in a 15% increase in mean total expenditures in the year prior to randomization. This substantial change in expenditures by a few participants supports the fact that these are true outliers.

## Discussion

In this analysis of a large care coordination program for low-income children and youth with chronic health conditions, overall Medicaid expenditures and utilization decreased considerably during the first year of the CHECK program for both CHECK participants and the UC group. Notably, expenditures did not increase among CHECK participants, which has been noted in other care coordination programs.^6^

Chronic disease among children has become common, yet we have a limited understanding of optimal methods of reducing health care expenditures. Theoretically, care coordination aimed at encouraging appropriate health care utilization should decrease expenditures over time. The CHECK program was designed to focus on decreasing expenditures and unnecessary health care utilization, with an emphasis on ED utilization. Additionally, the CHECK program ultimately focused efforts on participants with asthma and sickle cell disease and participants who had medium or high risk. Owing to the overall prevalence of asthma in the United States and the fact that the majority of CHECK participants had asthma, asthma management training was a substantial component of the care coordination team training. Similarly, owing to an established program for individuals with sickle cell disease at University of Illinois Health and Health Sciences System, the care coordination team received dedicated training about sickle cell disease. Although reducing all unnecessary health care utilization was a goal, the care coordination teams focused on preventing unnecessary ED utilization because ED utilization was high among CHECK participants. Thus, the observed changes in ED utilization among those considered medium to high risk and those with asthma or sickle cell disease may support the value of care coordination for these groups of children and young adults.

The lack of overall Medicaid savings in the first year after participation in CHECK may be explained by multiple reasons. First, CHECK was funded at the same time the state of Illinois rolled out managed care plans for Medicaid recipients in Cook County. During the first year of CHECK, the majority of publicly insured children and young adults in Cook County were transitioned from the traditional fee-for-service state Medicaid program to an MCO.^14,15^ This transition likely affected cost as MCOs manage cost, utilization, and quality of care differently than a fee-for-service program. Additionally, the transition to a Medicaid MCO may have disrupted care while beneficiaries attempted to navigate new insurance benefits and network restrictions. Statewide enrollment into Medicaid MCOs is a recent change in Illinois; however, this model has been used in other states and is often associated with cost savings, although findings have been inconsistent across states.^16,17^ Second, engaging families in care has been shown to result in increased utilization and expenditures,^7,17^ a finding that was not observed in our analysis. Third, most large care coordination programs have not resulted in cost savings, especially during the first year.^7,17^ The full benefit of care coordination may take years to occur, especially for a program focused on children and young adults.

### Limitations

Our study had limitations. First, this was a care delivery program; thus, the services provided to participants were based on participant needs and resources available and were not equal across all participants. This is different from traditional clinical trials, in which uniformity of exposure are a priority. Second, there are limitations to administrative claims data, including the risk of misclassification of diagnoses, miscoding, or duplicate claims. Third, identification of CHECK eligible participants was conducted by the individual Medicaid MCO using the eligibility criteria provided by CHECK; however, some participants did not meet all CHECK eligibility criteria (eg, no targeted CHECK diagnosis). This could be because of errors in the claims data or errors in how the payers identified eligibility. Fourth, CHWs are a growing but varied workforce, and the effectiveness of care coordination efforts likely varied by CHW as well.^18^

## Conclusions

Medicaid expenditures and utilization patterns decreased similarly among participants who were enrolled in the CHECK program and those in the UC group. Among children and young adults with asthma and those considered medium and high risk, a comprehensive care coordination program indicated that it could decrease ED utilization.

## Notes

Supplement 1. **Trial Protocol**

Supplement 2. **eTable 1. **Codes Used to Identify Specific Chronic Diseases for CHECK Eligibility **eTable 2. **Demographic Information of Outliers **eTable 3. **Mean Expenditures, Before and After Intervention, of Outliers **eFigure. **Mean Expenditures, Before and After Intervention, by 6-Month Increments, Showing Parallel Trends

Supplement 3. **Data Sharing Statement**
