# Supplementary material for: Effect of Comprehensive Care Coordination on Medicaid Expenditures Compared With Usual Care Among Children and Youth With Chronic Disease: A Randomized Clinical Trial
Source: JAMA Netw Open. 2019 Oct 4;2(10):e1912604. doi: 10.1001/jamanetworkopen.2019.12604 (PMC6784784; doi:10.1001/jamanetworkopen.2019.12604)
Supplement: Supplement 1. — Trial Protocol [file jamanetwopen-2-e1912604-s001.pdf]

Evaluation of the Coordinated Healthcare for Complex Kids (CHECK) Disease Interventions

Principal Investigator:
Molly A Martin, MD, MAPP
University of Illinois at Chicago
Department of Pediatrics
840 South Wood Street, M/C 856, Chicago, IL 60612
mollyma@uic.edu
312-996-2363

Co-Investigators:

- Benjamin Van Voorhees, MD: UIC Department of Pediatrics, bvanvoor@uic.edu
- Anne Elizabeth Glassgow, PhD: UIC Department of Pediatrics,aglassgo@uic.edu
- Rachel Caskey, MD: UIC Internal Medicine, rcaskey@uic.edu
- Mark Minier, MD: UIC Department of Pediatrics, mminier@uic.edu
- Daniel Touchette, PhD: UIC College of Pharmacy drtouche@uic.edu
- Andrea Pappalardo, MD: UIC Department of Pediatrics, apappa2@uic.edu
- Lewis Hsu, MD, PhD: UIC Department of Pediatrics, lewhsu@uic.edu
- Harsha Kumar, MD: UIC Department of Pediatrics, hvkumar@uic.edu
- Michelle Bulanda, PhD: UIC College of Applied Health Sciences, mbulanda@uic.edu
- Pam Roper, MD: UIC Miles Square Clinic, roperp@uic.edu
- Roxana Aguirre, MD: UIC Department of Pediatrics, raguirre@uic.edu
- Seema Awatramani, MD: UIC College of Medicine, seema@uic.edu
- Matthew Lucas, MD: UIC College of Nuring, lacasm@uic.edu

Study Locations:
University of Illinois at Chicago
Department of Pediatrics
840 South Wood Street, Chicago, IL 60612

Sponsor:
1C1CMS331342 Van Voorhees (Project Director)
Centers for Medicare and Medicaid Services, Health Care Innovation Awards
9/01/2014-8/31/2017
PAF: #2014-00701

Version: 1
Date: 05/18/2017

Table of Contents

		Page
1.0	Project Summary/Abstract	3
2.0	Background	3
2.1	Objectives/Aims	4
3.0	Eligibility	4
4.0	Subject Enrollment	5
5.0	Study Design and Procedures	5
6.0	Expected Risks/Benefits	5
7.0	Data Collection Management and Procedures	5
8.0	Data Analysis	6
9.0	Quality Control and Quality Assurances	6
10.0	Data Safety and Monitoring	6
11.0	Statistical Considerations	6
12.0	Regulatory Requirements	6
13.0	References	7
Appendix		9

Abbreviations

CHECK = Coordinated HEalthcare for Complex Kids
CHW = community health worker
UIC = University of Illinois at Chicago

1.0 PROJECT SUMMARY/ABSTRACT

University of Illinois CHECK (Coordinated HEalthcare for Complex Kids) is a quality improvement project that seeks to provide comprehensive, highly-innovative community-based care to children and young adults enrolled in Medicaid in Cook County, Illinois. CHECK is designed to improve care for populations with specialized needs, with a focus on the specific priority area of high-cost pediatric and young adult populations. CHECK has enrolled over 16,000 children and young adults ages 0-25 years old with diagnoses of asthma, diabetes, sickle cell disease, prematurity, mental health needs, oral health needs, or multiple health conditions. CHECK program enrollees are identified through their Medicaid insurance provider. Enrollees receive a variety of services that include access to a webportal where they can view and engage in educational activities, access to mental health promotion services, and community health worker (CHW) services. Services target general needs (transportation, access to care), diseases-specific needs, oral health promotion, and mental health promotion. CHW roles include patient enrollment, individual patient education and care coordination, and the creation of a “medical neighborhood” with community partners.

The goal of this IRB protocol is to evaluate the process and fidelity of CHECK services delivery, to determine associations between CHECK services and outcomes, and to disseminate these results.

2.0 BACKGROUND/SCIENTIFIC RATIONALE

In order to maintain optimal health and manage chronic medical conditions, children with chronic diseases and their families require comprehensive and coordinated services along the continuum of care to manage their complicated medical needs and facilitate transitions across providers and care settings.(1-3) However, providing this type of care to children with chronic diseases is extremely challenging,(4-6) and often these children receive substandard care that is uncoordinated, fragmented, and crisis-driven.(7,8) Consequently, this leads to a risk of poor health outcomes,(9) particularly for children living in poverty,(10,11) and increased emergency department visits, hospitalizations, and hospital readmissions.(3,5) In response to the high financial cost associated with caring for these children and recognition of the limitations in quality and availability of care, innovative models of care are emerging to lower the cost and offer higher quality services to children with chronic diseases and their families. Best practice standards of care have not been fully identified nor has a single model of health care delivery.(1) However, existing models represent the medical home theoretical concept and have similar foci on improved communication between patients, caregivers, and providers; improved access to specialized services and providers; and delivery of comprehensive and well-coordinated care.(20)

The value of improved quality of care is twofold: first, better health and overall well-being for most children with chronic diseases and their families, and secondly, less use of health care resources that result in reduced cost for payors.(2) Studies evaluating the medical home models for these children have a number of limitations including weak designs, inconsistent definitions of medical home attributes, and inconsistent outcome measures.(7) Despite these limitations, the findings have revealed a positive relationship between the medical home and improved outcomes, timeliness of care, family centeredness, and improved family functioning.(2) Although limited, the economic evidence also is promising that enrollment in specialized health care

programs designed for children with chronic diseases reduces overall health care costs.(12-18) Undoubtedly, there is need for the development and evaluation of additional health service delivery models for these children that improve the quality of their care while reducing the overall health care cost. The CHECK program is a comprehensive model of health care delivery that expands previous models and is designed to addresses the unique needs of children with chronic diseases and their families. The core financial principle of CHECK is to create incentives along with a funding mechanism to invest in non-reimbursable services (e.g., community health workers) that have been demonstrated to be effective in managing the care of populations with multiple chronic diseases, especially in underserved areas.

It is anticipated that the investment in additional support services to children with chronic diseases will result in improved and well-coordinated care that leads to a reduction of unnecessary inpatient hospitalizations, readmissions, and emergency department visits and a total cost savings. The CHECK quality improvement program has the potential to inform future cost-effective health care models aimed at improving the quality of life and care for children with chronic diseases and their families.

2.1 Objectives/Aims

The goals of this evaluation are to:

1. Describe the implementation of the CHECK disease specific interventions (diabetes, sickle cell disease, asthma, prematurity, mental health, and multiple chronic disease diagnoses).
2. Determine associations between CHECK services, disease and behavioral outcomes, and cost evaluations.

3.0 Eligibility

This evaluation targets enrolled CHECK participants. No recruitment of patients is done for this evaluation. CHECK families are already enrolled in CHECK.

3.1 Inclusion criteria

The only inclusion criteria for this protocol are participants must be an enrolled CHECK participant. Eligibility criteria for the CHECK quality improvement program include: 1) patients age 25 years or younger at the time of enrollment, 2) are enrolled in traditional Medicaid (fee-for-service) or one of the partner managed care plans, and 3) have a diagnosis of asthma, diabetes mellitus (type 1 or 2 if on daily diabetes-related medication), sickle cell disease, or prematurity (discharged from neonatal intensive care unit less than six months from date of enrollment).

3.2 Exclusion criteria

There are no exclusion criteria. If participants meet the inclusion criteria, they qualify.

3.3 Excluded or vulnerable populations

There are no excluded populations. The vulnerable populations include children (including neonates) and the economically disadvantaged.

4.0 Subject Enrollment

CHECK participants are already enrolled in CHECK which is a quality improvement project. No enrollment is done for this protocol.

5.0 Study Design and Procedures

5.1 CHECK Model

The CHECK Theoretical Model and CHECK Organizational Chart are in the Appendix. There are six teams within CHECK: community engagement, technology & data, mental health promotion, provider support services, care coordination, and legal services. Each of the teams has one or several directors that oversee services and staff.

5.2 CHECK Services

Services are provided to CHECK participants and their families according to the CHECK quality improvement project. Services include: primary care, specialty care, care coordination services, mental health promotion team services, legal services, and dental services. The amount of services are determined by the participant's risk category. The CHECK Risk Category assignment protocol, Intervention Protocol, and Disease Specific Protocols are also in the Appendix.

5.3 CHECK Data

Data are both retrospective and prospective, see section 7.0. There are no data requests or procedures that are specific to this protocol; the data are all already collected as standard of care for CHECK.

5.4 CHECK Participants

Participants were enrolled into the CHECK program beginning on December1, 2014 and enrollment is ongoing through August 31, 2017 or longer if a no-cost extension is granted by CMS. At the time that CHECK funding ends, CHECK services for enrolled families will either end or be transitioned to another funding source. CHECK participants that have been reached by CHECK staff are termed “engaged”. All participants are “enrolled”.

The CHECK funder, Centers for Medicare & Medicaid (CMS), contracted with Mathematica Policy Research to provide assistance in evaluating the CMS Innovation Awards. As part of the evaluation, Mathematica randomly assigns patients from one of the managed care organizations (MCOs) to receive either the CHECK intervention arm plus usual care or just usual care (Harmony Health Plan). To date, it has not been determined if CHECK services will be offered to the Harmony Health Plan patients at some later date.

6.0 Expected Risks/Benefits

CHECK will not release identifiable information on CHECK participants and therefore the risk is minimal. There are no direct benefits expected for the participants in regards to this evaluation.

7.0 Data Collection Management and Procedures

** Note that all data are already collected by the CHECK project.*

7.1 Types of Data

CHECK data is described in Table 1 and includes the type of data collected, source of the data, and the population in which the data is collected in CHECK. Data is collected, stored, and maintained by the CHECK Data Team.

Table 1. CHECK Data Sources.

Data Type	Source	Population
Claims (Cost/Utilization)	State HFS	CHECK Enrolled
Assessment Database - Tier1	Consensus MySQL	CHECK Engaged
Assessment Database - Tier2	Consensus MySQL	CHECK Engaged
Assessment Database - Tier3	Consensus MySQL	CHECK Engaged
Touches (Contacts)	Consensus MySQL	CHECK Engaged
Attendance	Chicago Public Schools (CPS)	CPS CHECK Patients
Enrollment Database	REDCap MySQL	CHECK Enrolled
ADT Feed	UI Health System	UIHP CHECK Enrolled
Social Service Referrals	Purple Binder	CHECK Engaged
SMS Logs	MyTapp/Onereach/Faer	CHECK Engaged
Self-Education Module Usage	Lifeguide	CHECK Enrolled
UIC Electronic medical records	Cerner	UIHP CHECK Enrolled

-

- The Illinois Department of Healthcare and Family Services (HFS) is the central resource for the claims data, which includes the Medicaid population characteristics, utilization, and payments. The CHECK program receives the data set monthly. .
- Consensus is the care coordination software CHECK uses to manage care coordination services. It is maintained in a MySQL database.
- Attendance data are received monthly from Chicago Public Schools (CPS). These data and analyses of these data are covered by a separate IRB protocol (2017-0404).
- Enrollment data are kept in a UIC REDCap database.
- An ADT feed is Admissions/Discharges/Transfers data which is received from UI Health Systems. The ADT feed is real time from the hospital production environment. It is stored in MYSQL Database. All messages are purged after 90 days of receipt.
- Social services referrals occur through an online program called Purple Binder which allows calculation of searches.
- SMS logs are number of text messages CHECK participants receive and respond to. These texts operate out of several systems: MyTapp, Onereach, and Faer.
- Self-education modules are online programs that participants can review to learn more about their disease. They are managed through a program called Lifeguide.
- The electronic medical records (EMR) of CHECK participants in the UH Health System will be accessed to gather clinical data such as blood or other tests, anthropometric values, and other clinical data as pertinent to analyses. These are collected through Cerner.

7.2 Data Storage

RedCap is an IRB approved web data collection tool. The REDCap server is behind a firewall, has virus protection, uses Secure Socket Layer authentication to encrypt communication between a user and the server, and has been configured to meet campus HIPPA rules. This means that the Internet connection between a user’s browser and

REDCap is encrypted, but REDCap is not encrypted on the server on which it resides, nor is any data downloaded and saved to a computer or other storage device encrypted unless a user takes steps to encrypt the stored file using third party software. All data collected through RedCap is stored on a secured MySQL database server. The database server does not have an externally translatable IP address and access to the server is controlled by Microsoft Active Directory. Permission controls and passwords will assure that only authorized personnel will have the ability to access study data. Account creation to the Redcap web server is managed by the Center for Clinical and Translational Sciences, project access is controlled by the project manager. The MRC will work with study staff to ensure accurate transfer of data to the MRC.

The Medicaid and CPS data are transferred from Medicaid/CPS to the UIC Center for Research Informatics (CRI). CRI runs a MySQL database on an RedHat Enterprise Linux 6 system protected by several layers of network security measures and encrypted to protect data in the event of hard drive theft. The network is protected by the UIC firewall at the outermost layer of network security, where the datacenter is on its own vLan. Access to the database from outside requires VPN tunneling through the UIC network into this particular vLan, which can only be done with UIC's permission. The virtual server that runs the database is also running its own iptables firewall that restricts all traffic to the local network. Finally, the MySQL database itself imposes access rules upon the users allowing access only from the local network. The datacenter is in a secure building allowing keycard access only. The folder that the MySQL database files are located in is encrypted with eCryptfs, rendering the database data files unreadable to those that do not have the passphrase. The SFTP server also enforces a set of strict policies to ensure data security while in transit and at rest. Login access restricted to the use of SFTP; other protocols including FTP, FTPS, and SCP are not allowed for external users. The SFTP server requires 2048 bit RSA Key authentication. Passwords based authentication is not allowed. The firewall only allows access to the local network and certain specific external IP address which external users must provide in the formal account request application. Encrypted file system for file storage and strict file subsystem isolation is enforced for all users. Each user’s home folder is located in a “chroot environment” so that their root directory is their home folder and the system-level file system is inaccessible to SFTP users.

7.3 Data Protections

The CHECK Data Team is responsible for all aspects of the protection of CHECK data, including protections of identifiable patient information. The CHECK data team will provide the requested data to authorized users in either .CSV or .XLSX universal formats that will be imported into SAS, SPSS, STATA, or other data analysis software. For any transfer of data, an encrypted file transfer program will be used to transfer onto a password protected UIC computer accessible only to the investigator. When possible, person identifiers will be replaced in the analysis file with a code linking them to the identifier and this code/identifier key will be kept in a separate file.

8.0 Data Analysis

The initial analysis is to tabulate basic summaries of the data collected. More sophisticated analyses may be indicated to identify predictors, mediators, and moderators of outcomes.

9.0 Quality Control and Quality Assurances

The data will be checked by investigators and the CHECK data analysis team to ensure quality control. This will be done every 3 months or as needed.

10.0 Data Safety and Monitoring

The research proposed presents only minimal risk as the data and procedures are already standard of care for CHECK and most data will be de-identified. If problems occur, they will be addressed immediately with the CHECK data analysis team and CHECK Publications Committee.

11.0 Statistical Considerations

To analyze each of the CHECK outcome,s univariate exploratory data analysis including measures of central tendency (means and medians) and measures of variability (range and standard deviations) will be calculated for continuous variables. Frequencies will be calculated for categorical variables. Multiple types of bivariate relationships also will be analyzed to determine the relationships between the variables. The methods for hypotheses testing will be based on specific CHECK outcomes.

12.0 Regulatory Requirements

12.1 Informed consent

We are requesting a waiver of informed consent. CHECK participants are already enrolled and cannot provide informed consent now for an evaluation of the tracking of the intervention they are already receiving.

12.2 Subject confidentiality

This is described in detail in the data collection and protocols section. Section 8.0 and Section 9.0.
CHECK data are kept for 5 years after the completion of the project.
Some data will be identifiable and data from CHECK participants will be used without informed consent because the goal of this research is to evaluate and disseminate the procedures and results of the interventions in this large quality improvement project.

12.3 Unanticipated problems

Any problems will be reported to the CHECK Publications Committee and/or IRB by the study investigators. If determined necessary, problems will also be reported to the funder.

13.0 References

1. Cohen E, Berry JG, Camacho X, Anderson G, Wodchis W, Guttmann A. Patterns and costs of health care use of children with medical complexity. Pediatrics. 2012;130(6):e1463-e1470.
2. Berry JG. What Children with Medical Complexity, Their Families, and Healthcare Providers Deserve from an Ideal Healthcare System. system. 2015;3:8.
3. Morin MJ, Alvey J, Murphy N, Glader L. Models of Care for Children with Medical Complexity. Health Care for People with Intellectual and Developmental Disabilities across the Lifespan: Springer; 2016:195-208.
4. Berry JG, Hall M, Neff J, et al. Children with medical complexity and Medicaid: spending and cost savings. Health Affairs. 2014;33(12):2199-2206.
5. Kingsnorth S, Lacombe-Duncan A, Keilty K, Bruce-Barrett C, Cohen E. Inter-organizational partnership for children with medical complexity: the integrated complex care model. Child: Care, Health and Development. 2015;41(1):57-66.
6. Matlow A, Wright J, Zimmerman B, Thomson K, Valente M. How can the principles of complexity science be applied to improve the coordination of care for complex pediatric patients? Quality and Safety in Health Care. 2006;15(2):85-88.
7. Berry JG, Agrawal RK, Cohen E, Kuo DZ. The landscape of medical care for children with medical complexity. Overland Park: Children's Hospital Association. 2013.
8. Sobotka SA, Hird-McCorry LP, Goodman DM. Identification of Fail Points for Discharging Pediatric Patients With New Tracheostomy and Ventilator. Hospital Pediatrics. 2016;6(9):552-557.
9. Gordon JB, Colby HH, Bartelt T, Jablonski D, Krauthoefer ML, Havens P. A tertiary care–primary care partnership model for medically complex and fragile children and youth with special health care needs. Archives of pediatrics & adolescent medicine. 2007;161(10):937-944.
10. Bona K, Blonquist TM, Neuberg DS, Silverman LB, Wolfe J. Impact of Socioeconomic Status on Timing of Relapse and Overall Survival for Children Treated on Dana-Farber Cancer Institute ALL Consortium Protocols (2000–2010). Pediatric blood & cancer. 2016.
11. Klitzner TS, Rabbitt LA, Chang R-KR. Benefits of care coordination for children with complex disease: a pilot medical home project in a resident teaching clinic. The Journal of pediatrics. 2010;156(6):1006-1010.
12. Casey PH, Lyle RE, Bird TM, et al. Effect of hospital-based comprehensive care clinic on health costs for Medicaid-insured medically complex children. Archives of pediatrics & adolescent medicine. 2011;165(5):392-398.
13. Cohen E, Lacombe-Duncan A, Spalding K, et al. Integrated complex care coordination for children with medical complexity: A mixed-methods evaluation of tertiary care-community collaboration. BMC health services research. 2012;12(1):1.
14. Palfrey JS, Sofis LA, Davidson EJ, Liu J, Freeman L, Ganz ML. The Pediatric Alliance for Coordinated Care: evaluation of a medical home model. Pediatrics. 2004;113(Supplement 4):1507-1516.
15. Silow-Carroll S, Hagelow G. Systems of care coordination for children: Lessons learned across state models. The Commonwealth Fund. 2010.
16. Innovative Services Delivery Models of Care. The CMS Innovation Center. 2016.
17. Illinois Community assessment of needs (UI-CAN) 2016: Toward health equity. 2016.
18. Coleman K, Austin BT, Brach C, Wagner EH. Evidence on the chronic care model in the new millennium. Health affairs. 2009;28(1):75-85.

APPENDIX Table of Contents	
**Appendix**	**Page Number**

Appendix	Page Number
CHECK Theoretical Model	11
CHECK Organizational Chart	12
CHECK Risk Categories	13
CHECK Intervention Protocol	14
Asthma Community Health Worker Protocol	15
Prematurity Community Health Worker Protocol	19
Sickle Cell Disease Community Health Worker Protocol	26
Mental Health Model and Protocol	31

CHECK THEORETICAL MODEL

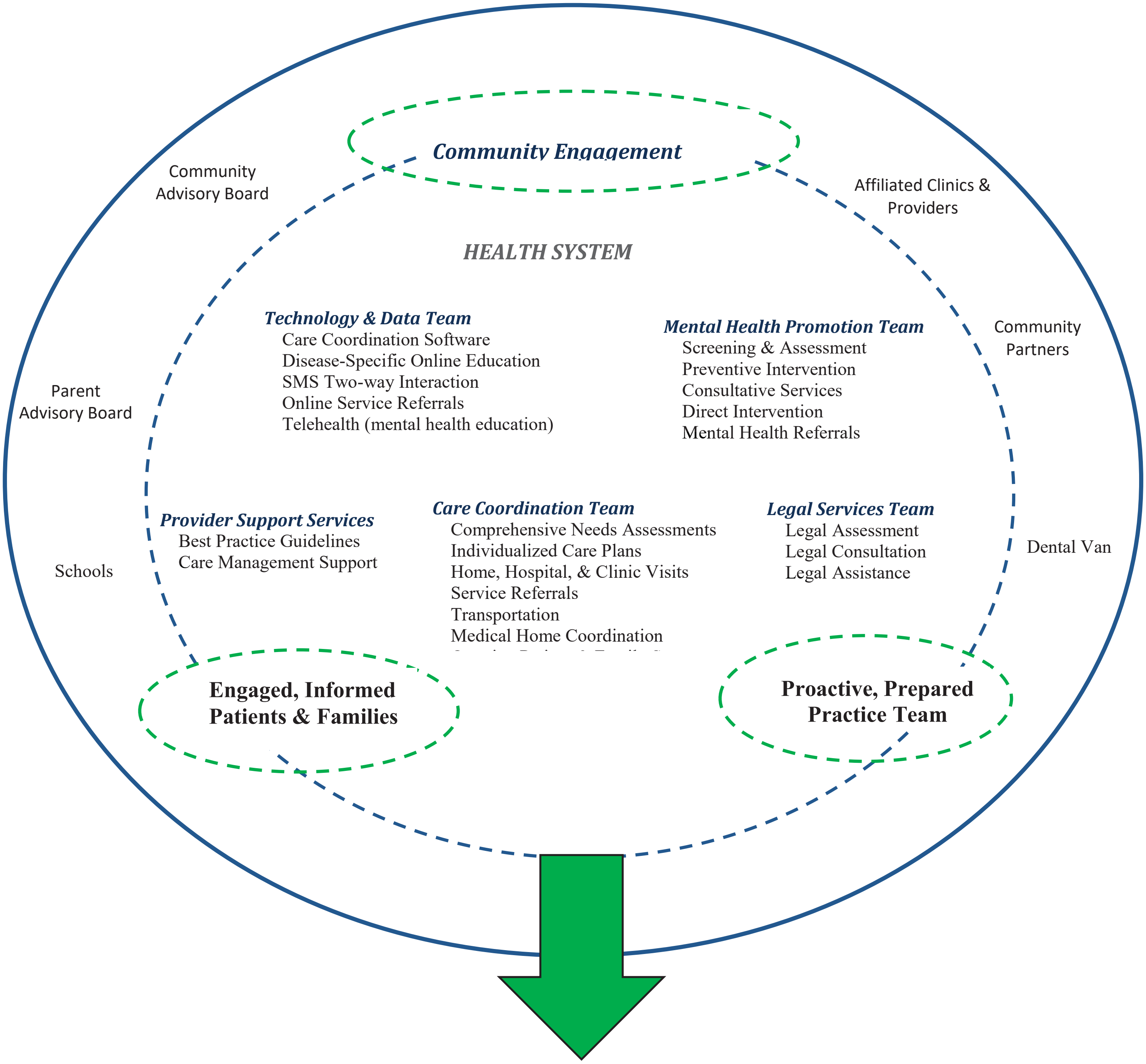

IMPROVED OUTCOMES

1. Reduced Health Costs
2. Reduced School absenteeism
3. Increased Patient Engagement

CHECK ORGANIZATIONAL CHART FOR PATIENT CARE

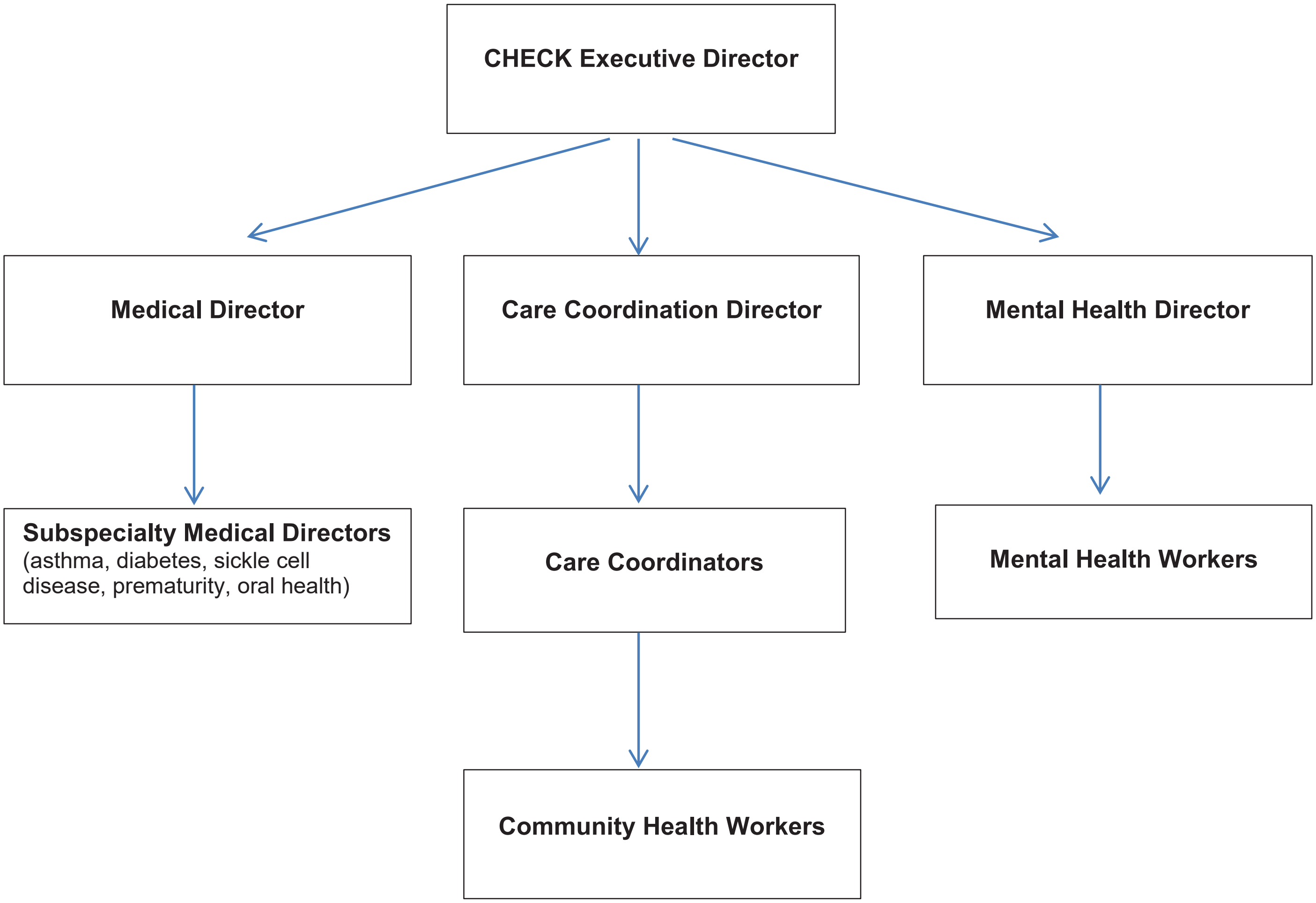

CHECK RISK CATEGORIES

	Claims Only
Low Risk	• No ED in last 12 months<br>• No hospitalization in last 12 months
Medium Risk	• 1-3 ED in last 12 month<br>*AND/OR*<br>• 1 hospitalization in last 12 months
High Risk	• >3 ED in last 12 month<br>*AND/OR*<br>• >1 hospitalization in last 12 months

To determine if disease *CONTROLLED*:

- **Asthma**: 20 or higher on Asthma Control Test or Childhood Asthma Control Test *(problem with the Childhood one – supposed to be administered visually to child, need to work out)*
- **Diabetes**:
  - Type 1 diabetes patient: HbA1c: <9.0%
  - Type 2 diabetes patient on insulin and oral medications: HbA1c<9.0%
  - Type 2 Diabetes patient on oral medications only: HbA1c<8.0%
- **Sickle cell**: Defined by severity, not control. Low severity =
  - <3 medical visits/yr for sickle cell pain
  - <2 episodes of sickle cell acute chest syndrome (ACS) lifetime
  - No new ischemic stroke in past year
  - No CKD (macroalbuminuria or GFR >60)
- **Prematurity**:
  - If meet CHECK inclusion criteria for prematurity, assume not well controlled for first 12 months.
  - At 12 months, controlled if
    - No ED/hospitalizations for respiratory issues in past 6 months
    - No failure to thrive
    - Is receiving Early Intervention services

CHECK INTERVENTION PROTOCOL

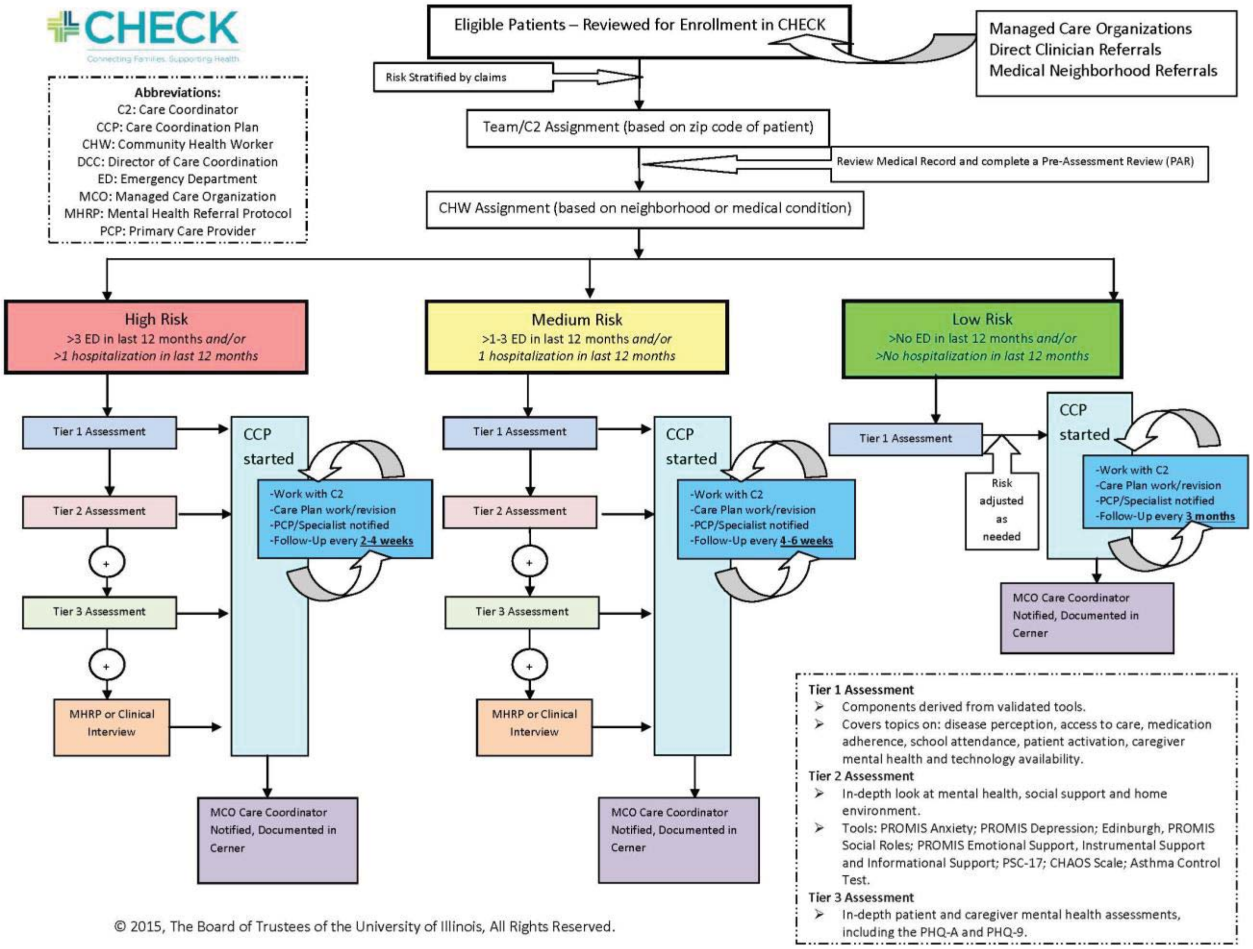

© 2015, The Board of Trustees of the University of Illinois, All Rights Reserved.

ASTHMA COMMUNITY HEALTH WORKER PROTOCOL

GOALS OF CHW INTERVENTION

1. Reduce missed doctor/health care provider appointments
2. Reduce missed days of school/work
3. Identify and reduce patients' barriers to medical care
4. Empower patients to take care of themselves as much as possible
5. Reduce avoidable/preventable hospitalization and ER visits
6. Help patient find and use appropriate medical resources
7. Improve patient satisfaction
8. Improve patient quality of life
9. Improve staff satisfaction
10. Ensure families know how and when to use asthma medications
11. Ensure families know how to reduce and avoid their asthma triggers

CHW CHARACTERISTICS FOR THIS POSITION

- People-oriented
- Experience with asthma preferred: asthma patient, relative/caregiver of a child/adult with asthma, retired healthcare professional

SKILLS FOR THIS POSITION

- Excellent communication skills
- Ability to work with individuals with a wide range of cognitive disabilities
- Ability to work with adolescents

RESOURCES NEEDED

Reliable cell phone
Personal transportation

RESPONSIBILITIES

1. Coach/teach patients and families how to improve the healthcare they receive
    a. Make and keep medical appointments
    b. Fill prescriptions in a timely fashion
    c. Learn how to monitor and report symptoms
    d. Understand medical decisions
    e. Ask questions
    f. Understand their health insurance requirements
2. Coach/teach patients and families how to avoid going to the ER or being hospitalized
    a. Make healthy asthma lifestyle choices and avoid risky behaviors
    b. Understand medical instructions
    c. Understand the difference between medically sound and unsound treatment
    d. Prevent or manage other medical conditions
3. Coach/teach patients and families to manage the disease
    a. Avoid asthma triggers
    b. How to medically manage exacerbations
    c. When to go to the clinic or hospital
    d. Alternatives to the ED and hospital
4. Coach/teach patients and families to use community resources

    a. Provide a list of resources
    b. Requests for reasonable accommodations at school or work
5. Communicate regularly with medical team and patients
    a. Report to medical staff anything unusual or of concern that requires action or information
6. Record data
    a. Record all encounters in Consensus
    b. Monitor care plan in Consensus
    c. Update care plan in Consensus
    d. Work with care coordinator to review Cerner encounters and add updates into Cerner on CHW activities
7. Supervision and ongoing support
    a. Report to care coordinator
    b. Attend CHECK general care coordination team meetings

TRAINING

Complete

- CHECK Basic Training
- CHECK Asthma Training
- 10 hours of observation in pulmonary/asthma clinic
- 10 hours of observation of asthma CHWs in the field (from CHICAGO Trial or Sinai)

PROTOCOL

FREQUENCY of Contacts

a. Routine contacts depend on risk level – *should be generated by Consensus*
    i. CHECK high risk
        1. CHW contacts as needed, minimum monthly but aim for every 2 weeks in person and/or phone
        2. If unable to reach a patient who is identified as high risk by telephone, the CHW will make a home visit to the last available address to attempt contact or obtain more current contact information. If unable to find the patient, the CHW will flag the patient in the CC and Cerner systems so that any future contact with UIH can be used to obtain more current information.
        3. Missed appointments will result in a CHW telephone call within 2 business days.
    ii. CHECK medium risk
        1. CHW contact phone/in person monthly
        2. Missed appointments will result in a CHW telephone call within 2 business days.
    iii. Low risk
        1. Won't qualify for specialty CHW
b. If hospitalized patient
    i. See hospital protocol. Goal is to visit patient in hospital minimum of once, aim for twice, discharge using CHECK hospital discharge form, and phone or in person follow-up within two days of discharge.
c. If patient in ED

    i. See ED protocol. Visit patient in ED if possible. Phone or in person follow-up with two days of ED visit.

VISIT Protocol

1. <u>Preparation for patient contact</u>
    a. Review information for patients you will assist.
    b. Organize important data
        i. Healthcare professionals involved in the patients' care: PCP, specialist
        ii. Departments, clinics involved in patients' care
        iii. Barriers, problems, issues that may have been noted in the past.
        iv. Patients' next appointments

2. <u>First contact</u>
    a. Contact the patients/caregivers by phone or by mail (contact with patient and caregivers will usually be in-person in the clinical setting or by telephone (voice and/or text messages).
    b. Introduce yourself as a CHW who been assigned by UIC to be their assistant.
    c. Set up plan for when and how you will have your next main visit

3. <u>First meeting</u>
    a. GOAL: Assess challenges and assets, develop a patient-driven work plan
    b. Be an active listener.
        i. Don't interrupt, but ask questions.
        ii. Take notes to show that you think what s/he is saying is important. Start a conversation
    c. Get to know the patient
        i. Talk a little about yourself.
            1. How long you've been doing this work
            2. Why you like your job
            3. Any personal information you want to share
        ii. Ask simple, not prying, questions about the patient and the caregiver
            1. Number of siblings, ages, grades
    d. Assess challenges and assets
        i. What makes asthma hard for you
        ii. Who or what helps you
        iii. Consider probing about transportation, scheduling, access to care team, communication with care team, finances (work limitations, healthcare costs), living situation
        iv. Assess how much they understand about asthma (symptoms, management, medications)
    e. Develop strategy/work plan
        i. Ask the patient and the caregiver what is the problem they would like to work on first with you. Might need to look at list from previous discussion and prioritize.
    f. Direct resources or support provision
        i. If relevant, assist patient with something. i.e. help them set up transportation or make an appointment, etc. (Otherwise ok just to make a plan and work on things bit by bit in the future.)

g. Action plan
    i. Make a written action plan with family. (This is not the same as an Asthma Action Plan from their provider. The Asthma Action Plan from the provider is an asthma-specific treatment plan. The CHECK action plan is a plan for behavior change the family sets.)
    ii. Give them copy
h. Set up next visit
    i. Set date and time
    ii. Ensure they have your contact info and you have best contact info for them.
i. Documentation
    i. As soon as possible (within 12 hours), document encounter in Consensus
    ii. Update care plan

4. <u>Subsequent meetings</u>
    a. Goal: To implement care plan
    b. Be an active listener.
        i. Don't interrupt, but ask questions.
        ii. Take notes to show that you think what s/he is saying is important. Start a conversation
    c. Review action plan from previous visit
        i. What worked, what didn't, encourage and revise
    d. Address 1-2 topics from Core Curriculum and/or care plan
    e. Make a new action plan (or review changes from previous that now become that day's action plan) Give them copy
    f. Set up next visit
        i. Set date and time
        ii. Ensure they have your contact info and you have best contact info for them.
    g. Documentation
        i. As soon as possible (within 12 hours), document encounter in Consensus
        ii. Update care plan

PREMATURITY COMMUNITY HEALTH WORKER PROTOCOL

GOALS OF CHW PROGRAM
1. Reduce missed outpatient appointments
2. Reduce missed days of school/work
3. Identify and reduce caregivers' and patients' barriers to care
4. Empower caregivers' and patients to take care of themselves as much as possible
5. Reduce avoidable hospitalization and ER visits
6. Help caregivers and patient find and use resources
7. Improve caregiver and patient satisfaction
8. Improve caregiver and patient quality of life
9. Improve staff satisfaction

CHW CHARACTERISTICS FOR THIS POSITION
- People-oriented
- Experience with prematurity preferred: relative/caregiver of a child/adult born prematurely, pervious healthcare professional
- Experience with community engagement and cultural tailoring

SKILLS FOR THIS POSITION
- Excellent communication skills
- Ability to work with individuals with a range of cognitive disabilities
- Ability to work with adolescents
- Excellent consumer service and be personable

RESOURCES NEEDED
Reliable cell phone
Personal transportation

RESPONSIBILITIES

1. Coach/teach patients and families how to improve the healthcare they receive
    a. Make and keep medical appointments
    b. Fill prescriptions in a timely fashion
    c. Describing symptoms
    d. Understand medical decisions
    e. Understand and receive immunizations
    f. Ask questions
    g. Understand their health insurance requirements
    h. Discharge Summary and passing on the information to other providers
2. Monitor utilization (Consensus and Cerner, with C2)
    a. Track attendance at medical appointments
    b. Track ED visits
    c. Track hospital days
3. Communicate regularly with medical team and caregivers
    a. Participate in medical team meetings where patients are discussed
    b. Submit written reports/EMR submissions as requested
    c. Use text or email to report to medical staff anything unusual or of concern that requires action or information
    d. Contact patient or family once per week
4. Coach/teach caregivers how to manage appointments (navigate outpatient system)
    a. Track number of consultations needed (primary care and subspecialties)
    b. Prioritizing appointments
    c. Scheduling different appointments (dates and times)
    d. Directions to appointments
    e. Travel needs and/or assistance
    f. Provide reminder calls
5. Coach/teach caregivers how to navigate insurance
    a. How to get on appropriate plans
    b. How to realize benefits
    c. Getting baby on medical card asap
6. Coach/teach caregivers how to manage transportation (navigate transportation systems)
    a. Review list of available resources
    b. Review pre-planning and scheduling strategies
7. Coach/teach caregivers how to avoid going to the ER or being hospitalized
    a. Understand medical instructions
    b. Understand the difference between medically sound and unsound treatment
    c. When to go to the Acute Care Center
    d. Alternatives to the ED and hospital
    e. Prevent or manage other medical conditions
8. Coach/teach caregivers to manage Prematurity special considerations
    a. CPR for acute situations (see referred videos)
    b. Understand baby poop cycle
    c. Track and assess developmental milestones
    d. Feeding needs
9. Coach/teach caregivers how to manage equipment
    a. Oxygen tank
    b. Monitor

    c. Feeding tube
    d. IV line
10. Coach/teach caregivers to use community resources
    a. Provide a list of resources
    b. WIC eligibility and process
    c. Preemie resources and support
        i. HealthyChildren.org
           141 Northwest Point Boulevard
           Elk Grove Village, IL 60007
           Phone: (847) 434-4000
           Web Address: www.healthychildren.org
        ii. www.grahamsfoundation.org
        iii. Illinois Premature Infant Health Network - EverThrive Illinois
           www.ilmaternal.org/ipihn/ Illinois Maternal & Child Health Coalition
11. Coach/teach caregivers how to track premature baby's developmental milestones
    a. Common preemie examinations
    b. Preemie Milestones: Ages and Stages
    c. Early intervention referral or other referrals when needed
    d. High Risk Clinic follow-up and referrals

PROTOCOLS

FREQUENCY of Contacts

a. Routine contacts depend on risk level – *should be generated by Consensus*
    ii. CHECK high risk
        1. CHW contacts as needed, minimum monthly but aim for every 2 weeks in person and/or phone
        2. If unable to reach a patient who is identified as high risk by telephone, the CHW will make a home visit to the last available address to attempt contact or obtain more current contact information. If unable to find the patient, the CHW will flag the patient in the CC and Cerner systems so that any future contact with UIH can be used to obtain more current information.
        3. Missed appointments noted through Consensus or Cerner (or patient self-report) will result in a CHW telephone call within 1 week (or as dictated by primary CHECK protocols)
    iii. CHECK medium risk
        1. CHW contact phone/in person monthly
        2. Missed appointments will result in a CHW telephone call within 2 business days.
    iv. Low risk
        1. Won't qualify for specialty CHW
b. If hospitalized patient
    v. See hospital protocol. Goal is to visit patient in hospital minimum of once, aim for twice, discharge using CHECK hospital discharge form, and phone or in person follow-up within two days of discharge.
c. If patient in ED
    vi. See ED protocol. Visit patient in ED if possible. Phone or in person follow-up with two days of ED visit.

VISIT Protocol FOR BABY STILL IN NICU

1. Preparation for patient contact
    a. Review information for patient you will assist.
    b. Organize important data
        i. Healthcare professionals involved in the patients' care: PCP, specialist
        ii. Barriers, problems, issues that may have been noted in the past.
        iii. Patients' anticipated discharge date
2. First contact
    a. Contact the patients/caregivers by phone or in person
    b. Introduce yourself as a CHW who been assigned by UIC to be their assistant.
    c. Tier 1 or 2 assessment if not done
    d. Set up plan for when and how you will have your next main visit

3. First meeting
    a. GOAL: Assess challenges and assets, develop a patient-driven work plan
    b. Be an active listener.
        i. Don't interrupt, but ask questions.

        ii. Take notes to show that you think what s/he is saying is important. Start a conversation
    c. Get to know the patient
        i. Talk a little about yourself.
            1. Brief, as much as comfortable with
        ii. Ask simple, not prying, questions about the patient and the caregiver
            1. Number of siblings, ages, grades
    d. Assess challenges and assets
        i. What makes having this baby hard for you
        ii. Who or what helps you
        iii. Consider probing about transportation, scheduling, access to care team, communication with care team, finances (work limitations, healthcare costs), living situation
        iv. Assess how much they understand about their baby (symptoms, management, medications)
    e. Develop strategy/review Care Plan
        i. Ask the patient and the caregiver what is the problem they would like to work on first with you. Might need to look at list from previous discussion and prioritize.
    f. Direct resources or support provision
        i. If relevant, assist patient with something. i.e. help them set up transportation or make an appointment, etc. (Otherwise ok just to make a plan and work on things bit by bit in the future.)
    g. Action plan (Not Care Plan. Action plans are small plans to make change between visits.)
        i. Make a written Action Plan with family.
        ii. Give them copy
    h. Set up next visit
        i. Set date and time
        ii. Ensure they have your contact info and you have best contact info for them.
    i. Documentation
        i. As soon as possible (within 12 hours), document encounter in Consensus
        ii. Update care plan
        iii. C2 to document periodic summaries in Cerner and when information could be clinically useful to UIC staff/providers

4. <u>Subsequent meetings</u>
    a. Goal: To implement care plan
    b. Be an active listener.
        i. Don't interrupt, but ask questions.
        ii. Take notes to show that you think what s/he is saying is important. Start a conversation
    c. Review Action Plan from previous visit
        i. What worked, what didn't, encourage and revise
    d. Address 1-2 topics from NICU Core Curriculum and/or Care Plan
    e. Make a new Action Plan (or review changes from previous that now become that day's Action Plan) Give them copy
    f. Update contact info
    g. Set up next visit

        i. Set date and time
        ii. Ensure they have your contact info and you have best contact info for them.
    h. Documentation
        i. As soon as possible (within 12 hours), document encounter in Consensus
        ii. Update Care Plan

VISIT Protocol FOR BABY DISCHARGED FROM NICU

1. <u>Preparation for patient contact</u>
    a. Review information for patients you will assist.
    b. Organize important data
        i. Healthcare professionals involved in the patients' care: PCP (visits should start 1-2 days after discharge), specialist
        ii. Departments, clinics involved in patients' care
        iii. Barriers, problems, issues that may have been noted in the past.
        iv. Patients' next appointments

2. <u>First contact</u>
    a. Contact the patients/caregivers by phone or by mail (contact with patient and caregivers will usually be in-person in the clinical setting or by telephone (voice and/or text messages).
    b. Introduce yourself as a CHW who been assigned by UIC to be their assistant.
    c. If Tier 1 or Tier 2 assessment not completed, complete.
    d. Set up plan for when and how you will have your next main visit

3. <u>First meeting</u>
    a. GOAL: Assess challenges and assets, develop a patient-driven work plan
    b. Be an active listener.
        i. Don't interrupt, but ask questions.
        ii. Take notes to show that you think what s/he is saying is important. Start a conversation
    c. Get to know the patient
        i. Talk a little about yourself.
            1. Brief, as much as you feed comfortable
        ii. Ask simple, not prying, questions about the patient and the caregiver
            1. Number of siblings, ages, grades
    d. Assess challenges and assets
        i. What makes caring for this baby hard for you
        ii. Who or what helps you
        iii. Consider probing about transportation, scheduling, access to care team, communication with care team, finances (work limitations, healthcare costs), living situation
        iv. Assess how much they understand about prematurity (symptoms, management, medications)
    e. Develop strategy/review Care Plan
        i. Ask the patient and the caregiver what is the problem they would like to work on first with you. Might need to look at list from previous discussion and prioritize.
    f. Direct resources or support provision

        i. If relevant, assist patient with something. i.e. help them set up transportation or make an appointment, etc. (Otherwise ok just to make a plan and work on things bit by bit in the future.)

   g. Action plan (Not Care Plan. Action plans are small plans to make change between visits.)
        i. Make a written Action Plan with family.
        ii. Give them copy
   h. Set up next visit
        i. Set date and time
        ii. Ensure they have your contact info and you have best contact info for them.
   i. Documentation
        i. As soon as possible (within 12 hours), document encounter in Consensus
        ii. Update Care Plan

4. <u>Subsequent meetings</u>
   a. Goal: To implement Care Plan
   b. Be an active listener.
        i. Don't interrupt, but ask questions.
        ii. Take notes to show that you think what s/he is saying is important. Start a conversation
   c. Review Action Plan from previous visit
        i. What worked, what didn't, encourage and revise
   d. Address 1-2 topics from Prematurity Core Curriculum and/or Care Plan
   e. Make a new Action Plan (or review changes from previous that now become that day's Action Plan) Give them copy
   f. Update contact info
   g. Set up next visit
        i. Set date and time
        ii. Ensure they have your contact info and you have best contact info for them.
   h. Documentation
        i. As soon as possible (within 12 hours), document encounter in Consensus
        ii. Update Care Plan

SICKLE CELL DISEASE COMMUNITY HEALTH WORKER PROTOCOL

GOALS OF COMMUNITY HEALTH WORKER (CHW) INTERVENTION

1. Reduce missed doctor appointments, especially for these target groups:
    a. Adolescent/ young adult transition
    b. Chronic blood transfusion
    c. High risk for stroke - abnormal transcranial Doppler ultrasound
    d. Asthma or chronic lung disease
    e. Multiple hospitalizations for pain, spleen, or other sickle complications
    f. Infants up to 12 months old
    g. Chronic kidney disease
    h. Patients receiving hydroxyurea
2. Reduce missed days of school/work
3. Identify and reduce patients' barriers to care
4. Empower patients to take appropriate care of themselves as much as possible
5. Reduce **avoidable** hospitalization and ER visits
6. Reduce periods when prescription meds have run out
7. Help patient find and use resources
8. Improve patient satisfaction
9. Improve patient quality of life

CHW CHARACTERISTICS FOR THIS POSITION

- People-oriented
- Experience with sickle cell disease preferred: SCD patient, relative/caregiver of a child/adult with SCD, retired healthcare professional, member of sickle community group (such as SCDAI, SCDAA)

SKILLS FOR THIS POSITION

- Excellent communication skills
- Ability to work with individuals with a range of cognitive disabilities
- Ability to work with adolescents
- Ability to document encounters with patients/families and convey messages to care team
- Ability to interpret clinical visit information to patients/caregivers when needed

RESOURCES NEEDED

Reliable cell phone
Personal transportation

RESPONSIBILITIES

1. Coach/teach patients and families how to improve the healthcare they receive
    a. Make and keep medical appointments
    b. Fill prescriptions in a timely fashion
    c. Learn how to monitor and report symptoms
    d. Understand medical decisions
    e. Ask questions (don't leave the visit saying, "I wish I remembered to ask about that!")
    f. Understand their health insurance requirements
2. Coach/teach patients and families how to avoid going to the ER or being hospitalized
    a. Make healthy sickle cell disease lifestyle choices and avoid risky behaviors

    b. Understand medical instructions
    c. Understand the difference between medically sound and unsound treatment
    d. Prevent or manage other medical conditions
3. Coach/teach patients and families how to seek medical attention promptly for potentially serious medical complications
    a. Fever
    b. Difficulty breathing
    c. New problems with weakness, numbness or coordination that could be signs of a stroke
4. Coach/teach patients and families to manage the disease
    a. Avoid triggers for pain
    b. Tools for home pain management
    c. When to go to the Acute Care Center
    d. Alternatives to the ED and hospital
    e. Encourage compliance with hydroxyrea therapy
    f. Encourage compliance with transfusion program
5. Coach/teach patients and families to use community resources
    a. Provide a list of resources
    b. Requests for reasonable accommodations at school or work
6. Communicate regularly with medical team and patients
    a. Participate in medical team meetings where patients are discussed
    b. Report to medical staff anything unusual or of concern that requires action or information
7. Record data
    a. Record all encounters in Consensus
    b. Monitor care plan in Consensus
    c. Update care plan in Consensus
    d. Work with care coordinator to review Cerner encounters and add updates into Cerner on CHW activities
8. Supervision and ongoing support
    a. Report to care coordinator
    b. Attend CHECK general care coordination team meetings

TRAINING

Complete initial training

- CHECK Basic Training
- CHECK SCD Training
- Read “Hope and Destiny Jr.” book for teens with sickle cell, by Hsu, Brandalise, Rodrigues
- 7 hours (2 clinic sessions) of observation in sickle cell clinic

Continuing education

- SCD annual/semiannual update – Dr. Hsu
- SCDAI annual conference February, Oak Lawn, IL – this has a registration fee around $40
- 3.5 hr observation in sickle cell clinic how often (Kenita and Lewis to decide)

PROTOCOLS

FREQUENCY of Contacts

a. Routine contacts depend on risk level – *should be generated by Consensus*

vii. CHECK high risk

        1. CHW contacts as needed, minimum monthly but aim for every 2 weeks in person and/or phone
        2. If unable to reach a patient who is identified as high risk by telephone, the CHW will make a home visit to the last available address to attempt contact or obtain more current contact information. If unable to find the patient, the CHW will flag the patient in the CC and Cerner systems so that any future contact with UIH can be used to obtain more current information.
        3. Missed appointments will result in a CHW telephone call within 2 business days.
    viii. <u>CHECK medium risk</u>
        1. CHW contact phone/in person monthly
        2. Missed appointments will result in a CHW telephone call within 2 business days.
    ix. <u>Low risk</u>
        1. Won't qualify for specialty CHW
b. If <u>hospitalized patient</u>
    x. See hospital protocol. Goal is to visit patient in hospital minimum of once, aim for twice, discharge using CHECK hospital discharge form, and phone or in person follow-up within two days of discharge.
    xi. This might be a stressful & challenging event. Note how they respond and note any barriers.
c. If <u>patient in ED</u>
    xii. See ED protocol. Visit patient in ED if possible. Phone or in person follow-up with two days of ED visit.
    xiii. This might be a stressful and challenging event. Note how they respond and note any barriers.

VISIT Protocol

1. <u>Preparation for patient contact</u>
    a. Review information for patients you will assist.
    b. Organize important data
        i. Healthcare professionals involved in the patients' care: PCP, sickle cell team
        ii. Departments, clinics involved in patients' care
        iii. Barriers, problems, issues that may have been noted in the past.
            1. For high-risk and medium-risk: Direct discussion with SCD clinical team, chart review of social work and psychology notes
            2. For low-risk: From chart review
        iv. Patients' next appointments
2. <u>First contact</u>
    a. Contact the patients/caregivers by phone or by mail (contact with patient and caregivers will usually be in-person in the clinical setting or by telephone (voice and/or text messages).
    b. Introduce yourself as a CHW who been assigned by UIC to be their assistant.
    c. Set up plan for when and how you will have your next main visit

3. <u>First meeting</u>
    a. GOAL: Assess challenges and assets, develop a patient-driven work plan

b. Be an active listener.
    i. Don't interrupt, but ask questions.
    ii. Take notes to show that you think what s/he is saying is important. Start a conversation
c. Get to know the patient
    i. Talk a little about yourself.
        1. How long you've been doing this work
        2. Why you like your job
        3. Any personal information you want to share
    ii. Ask simple, not prying, questions about the patient and the caregiver
        1. Number of siblings, ages, grades
d. Assess challenges and assets
    i. What makes sickle cell hard for you
    ii. Who or what helps you
    iii. Consider probing about transportation, scheduling, access to care team, communication with care team,, do you know what number to call?, finances (work limitations, healthcare costs), living situation
    iv. Assess how much they understand about sickle cell disease (symptoms, management, medications)
        1. In particular, assess their understanding of emergency situations (fever of 101.4 F or 38.5 C, difficulty breathing, extreme pain, or signs of stroke)
        2. If less than perfect understanding, review FAST poster
e. Develop strategy/work plan
    i. Ask the patient and the caregiver what is the problem they would like to work on first with you. Might need to look at list from previous discussion and prioritize.
f. Direct resources or support provision
    i. If relevant, assist patient with something. i.e. help them set up transportation or make an appointment, etc. (Otherwise ok just to make a plan and work on things bit by bit in the future.)
g. Action plan
    i. Make a written action plan with family.
    ii. Give them copy
h. Set up next visit
    i. Set date and time
    ii. Ensure they have your contact info and you have best contact info for them.
i. Documentation
    i. As soon as possible (within 12 hours), document encounter in Consensus
    ii. Update care plan
    iii. For high-risk patients, provide a copy to Care Coordinator and SCD clinical team

4. <u>Subsequent meetings</u>
    a. Goal: To implement care plan
    b. Be an active listener.
        i. Don't interrupt, but ask questions.
        ii. Take notes to show that you think what s/he is saying is important. Start a conversation

c. Review action plan from previous visit
    i. What worked, what didn't, encourage and revise
d. Assess their understanding of emergency situations (fever of 101.4 F or 38.5 C, difficulty breathing, extreme pain, or signs of stroke)
    i. If less than perfect understanding, review FAST poster
e. Address 1-2 topics from Core Curriculum and/or care plan
f. Make a new action plan (or review changes from previous that now become that day's action plan) Give them copy
g. Set up next visit
    i. Set date and time
    ii. Ensure they have your contact info and you have best contact info for them.
h. Documentation
    i. As soon as possible (within 12 hours), document encounter in Consensus
    ii. Update care plan

CHECK MENTAL HEALTH MODEL& PROTOCOL

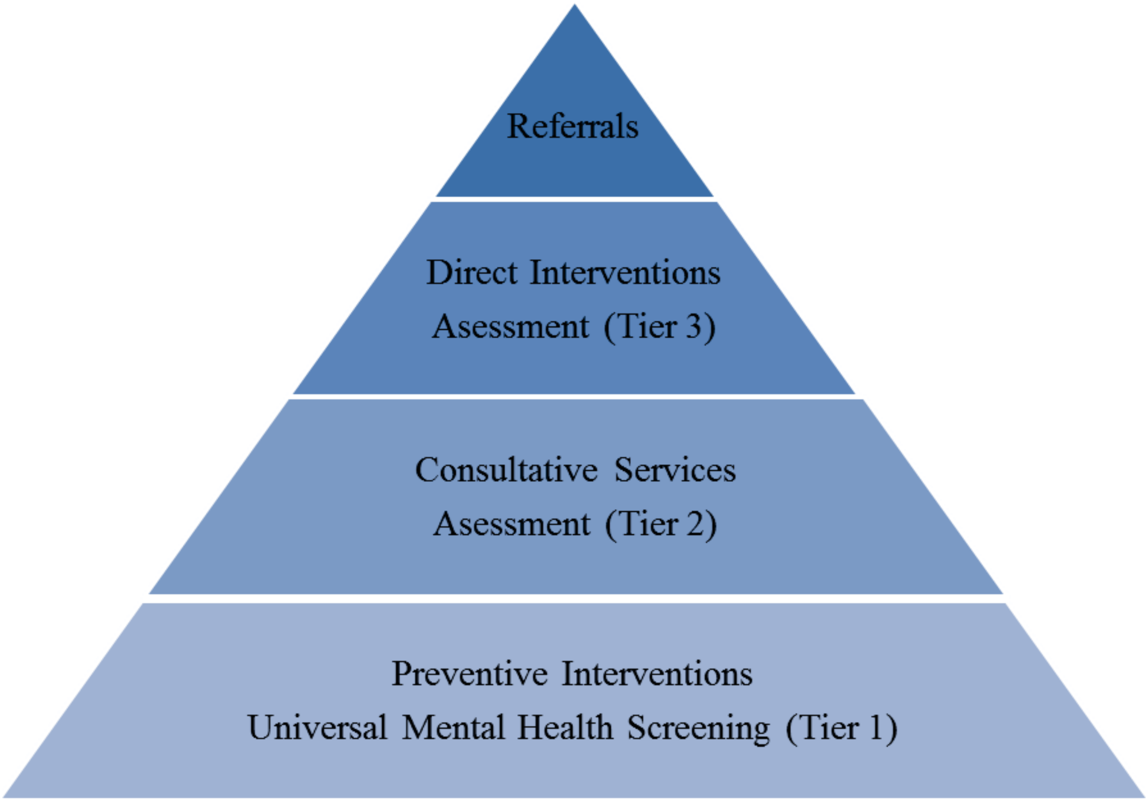

Figure 1. CHECK Mental Health Model.

Mental Health Protocol

I. **Background -** The promotion of mental health for children, young adults, and parents of children with medical illness (Sickle Cell Disease, Asthma, Diabetes, and Prematurity) is one objective of the CHECK program. This protocol will provide a guide to health promotion and wellness services offered through the CHECK program, as well as to various protocols staff will follow under certain circumstances.

II. **Overview -** There is a three tier process in place for CHECK MHP. Tier I and Tier II are typically conducted outside of the MHP team and provide a basis for determining whether an evaluation (Tier III) is needed by the CHECK MHP team. However, there are instances wherein members of the MHP team conduct Tier I and Tier II assessments. For example, if members of the MHP team make initial contact with CHECK participants in a clinic, then they will conduct the assessments. Additionally, other members are assisting with Tier I and Tier II assessments due to a surplus of assessments needing to be completed. Only participants who are considered to be medically high risk, who are experiencing mild to moderate behavioral concerns, or are the parent of a newborn will be eligible for MHP services within CHECK MHP. Those experiencing high risk mental health concerns (e.g., a history of suicidal thoughts, non-suicidal self-injury, risk for child abuse) will be given psychiatric referrals. Additionally, participants with a mental health disorder or severe mental health difficulties (e.g., Major Depressive Disorder, Post Traumatic Stress Disorder, Oppositional Defiant Disorder, or developmental disorders, psychotic symptoms, mania, substance abuse/dependence) will be provided with psychiatric referrals as they require a higher level of care. Based on the level of need, various resources can be offered to the participant following evaluation and scoring of measures to engage them in antecedent and response-focused emotion regulation as mechanisms for change. For teens under 20, communication will only be through a parent/guardian and they will not be assessed independently, unless parent consent is obtained and documented. Teens 20 and over can provide self-reported responses and will be expected to manage their own participation in the service.

III. **Qualification for Services -** Eligibility for MHP is determined by a comprehensive tiered assessment protocol (see Evaluation Protocol for detailed description of this process). Children, adolescents, families, and parents experiencing sub-threshold clinical symptoms may qualify for services. Participants experiencing more elevated symptoms or present with risk (e.g., suicidal thoughts) will be referred for outside services.

IV. Intake Protocol – An interview will be conducted with participants who qualify for CHECK mental health services and are interested in receiving these services. This can be done immediately following the Tier III Assessment or at a later time (to be scheduled upon completing the Tier III).

- Intake evaluation should take approximately 20-30 minutes.
- It involves a series of open ended questions regarding the family's social-emotional health as it relates to engagement in health care, and is meant to be completed in a semi-structured way
- For participants under 18, the parent or primary caregiver will be interviewed regarding their child.
- For young adults age 20 or over, all responses should be their own.
- Based on the participant's level of need, we may offer (details on each option provided in subsequent sections below):
    - Web-based modules
    - Telehealth
    - Direct, in person intervention at 840 S. Wood St. – schedule first appointment while on the phone with the participant if possible
- A referral for outside services should be given if further assessment via the intake interview suggests that the participant is struggling with mental health needs (e.g., a mental health disorder as defined by distress and impairment)
- Sample Script for Explanation of Services for which they qualify:
    - <u>Qualify for CHECK MHP (Level 1-3):</u> *Thank you for going through those questions with me and for sharing so much. Based on what you are letting me know about you/your child, it sounds as though we have some services here that might be helpful to you/your child. I will tell you a little about them and make sure you have the information you need at this time. Before we go on, do you have any questions for me?*
        - Answer any questions participants/parents may have
    - <u>If they do not qualify (Level 4):</u> *Thank you so much for going through those questions with me. It sounds as though right now you/your child are/is really struggling with XXX, and I can understand your concern and desire to help yourself/your child work on that. I want to help you best meet those needs, so instead of working directly with our group here, I would like to connect you with someone who can provide a more targeted/specialized approach to helping you/your child.*
        - Provide appropriate referral from Purple Binder
        - Inform the participant that their CHW will follow up with them regarding all of their CHECK services and can help them further with a referral if needed
        - Inform the participant that they can consult with their other doctors regarding their mental health needs if they have concerns moving forward

- Safety of Participants: If at any time you become aware of safety concerns for the child or a family member, follow the Suicide Protocol or contact appropriate authorities to notify them of the concern (911, DCFS, etc.)
    - Consult with a supervisor regarding all safety events or DCFS referrals.
    - If contacting DCFS: Gather as much information as possible regarding the names, ages, addresses, phone numbers, concerns (time(s), place(s), type of harm, who is harming whom, injuries/experiences, etc.)
        - Follow Abuse Protocol (see below) closely in the event that DCFS is contacted.
        - Also follow University of Illinois Hospital and Clinics Abuse Protocol (titled “Identification and Management of Victims of Abuse or Neglect”)
    - If there is a medical emergency that occurs during the call, be sure to also contact the participant’s physician and case manager to inform them of the event after contacting emergency services.
    - Stay on the phone with the participant for as long as necessary to ensure their safety and connection to in person care.
    - Do not offer to transport the participant to an emergency room/hospital
    - Do not offer medical advice

V. **Mental Health Referral Protocol** – If the participant does not qualify for services within CHECK, refer them to outside services through Purple Binder

- check.purplebinder.com
- Search via zip code and the services they need
- Call participant and give them the referrals
- Follow up in 1-2 weeks to see if they made contact and were able to initiate services
- If not, help facilitate this process.

VI. **Mental Health Promotion Services–** If the participants qualify for services within CHECK, we can offer several options (see below). CHECK MHP team members will provide these options based on the participant’s level of need, age, transportation, and personal preference (e.g., convenience of online modules).

- Web-based modules:
    - Provide them with the CHECK website (www.mycheck.uic.edu) and tell them they can access the online modules through the mental health portal).
    - Follow up in 1-2 weeks and see if they have signed into the website, if they have found the modules helpful, and if they have any questions.
    - Ask if there is anything else we can do for them
- Telehealth: This would allow participants to access services remotely from their home (see Telehealth Protocol for detailed information).

- VSee is a telemedicine platform that will be used for these services.
- Participants are provided a link via email, and they can "log in" by clicking this link
- Devices supported include Mac, Windows, and Android computers and smart phones
- Participants will then meet with MHP staff through this platform

- In person meetings: In person MHP services are currently being provided by provided. Supervision is received for two hours weekly (one hour of group supervision and one hour of individual supervision) to consult regarding MHP.
    - Extern will schedule first appointment
        - In the event that a participant indicates interest but then the extern is unable to reach to schedule:
            - Attempt to contact participant up to three times, averaging one phone call per week.
            - It is fine to leave a message on each attempt.
            - If we have permission, consider sending a text message with MHP team member's contact information.
            - After three phone calls/text messages, close the task in consensus and leave a note indicating that the participant can request services again in the future should they become interested.
            - Refer back to the CHW so they can initiate the Hard to Reach protocol if needed.
    - Provide participant with parking options (e.g., Wood parking structure, free parking, public transportation)
    - Tell participant to call extern on their direct line when they reach the building (840 S. Wood St. Chicago, IL, 60612). The extern will meet them in the lobby and take them to the consultation room
    - Direct intervention services are provided in a small conference room on the 14th floor. When necessary, the extern may check out a second room (e.g., two participants scheduled for the same time). This room is in a small hallway and privacy is maximized
    - Externs will determine if the participant has visited the UI Health System in the last year to determine if the hospital's consent to treat is valid. If it is not, then a consent to treat document will be signed at their first visit.
    - Toys and games are provided for child participants
    - The intake interview will determine the participants' needs for the services provided, which include pain management, coping strategies, parenting support groups, motivational interviewing, and mindfulness skills designed to help families struggling with chronic illness.
    - MHP sessions will be logged in Consensus. Session notes are to include:

        - Who was present
        - The focus of the session
        - Plan for the next session
        - Note regarding status of consent to treat document
        - Reasons for cancellations/no shows and action taken by extern in this event
    - Most participants will be seen between 3-6 times, with adjustments made on an as needed basis when there is significant reason to end/continue services outside of this window
    - In the event of participant no shows:
        - A phone call will be made to check in and reschedule; up to three phone calls can be made to attempt to reschedule within a three week period.
        - If we have permission, consider sending a text message with MHP team member's contact information.
        - After three phone calls/text messages, close task, note in consensus, and discontinue services.
        - Refer back to the CHW so they can initiate the Hard to Reach protocol if needed.
        - Participants may reinstate services in 2-3 months if they are interested.
        - After three no shows, the participant will be reminded that they have 6 sessions that they can use at a later date if they would like, but for now, we will help them obtain outside services if they would like.
        - Their services are discontinued and can be reinstated in 2-3 months.
- Safety:
    - If participants express any thoughts of suicide or self-harm, the Suicide Protocol will be followed.
    - If child abuse is suspected, the Child Abuse Protocol will be followed.

VII. **Suicide Protocol -** If a subject responds positively (endorsing 1 – 3) on the suicide question on the PHQ-9, the interviewer immediately transitions to the suicide protocol.

- Verify address of where they are at AND phone number prior to beginning suicide protocol/early on in the process in case they will not provide it later.
- Ask the following questions to assess risk:
    - *Have you had specific thoughts about how you might hurt yourself?*
    - *Do you think you might act on these thoughts?*
- If the participant answers yes to either of these questions, they are considered High Risk for the purposes of CHECK MHP and will be referred out/referred back

to their existing psych contacts. Goal in this situation is to recommend they call their primary care doctor or a mental health provider.

  - **FOR TEENS, first:** Facilitate them telling their parents while we listen in or facilitate the conversation for them (when teen is being seen in person).
  - **FOR ALL:** Get them help, via
    - Asking them to call their primary care provider (who can give them a referral), or a mental health provider if they have one
    - Give them a referral – Purple Binder (For UI Health + → Mile Square/Psychiatry)
    - Give them the suicide hotline number: 1-800-273-TALK (8255)
    - Tell them to reach back out to the CHECK team if they need any help/support with the previous options: Provide CHW's phone # (Notify CHW via Consensus)
  - Try to end the interview on a positive note. It is a good idea to bring the interview around to a more neutral topic or to discuss the kid's strengths.
  - Even when there is no immediate danger, consult with Dr. Jacobs as soon as possible if there is any immediate risk. This can be done with or without the subject in the room, depending on the circumstances. Explain to the subject that you need to consult with your supervisor whenever someone expresses suicidal thoughts.
  - Follow up with the child's C2 and CHW via consensus notes. "Link" siblings if applicable.
  - Dr. Jacobs should always be consulted when suicidal feelings are expressed; immediately or within 24 hours depending on the circumstances. In the rare instant where she cannot be available, please call the UIC psychiatry resident on call.
  - Follow up as needed to make sure linked with services.

- If yes to both items:
  - *Ask whether participant has access to their planned means? Are you safe?*
- If participant has access to means and has endorsed both questions, we:
  - **FOR TEENS, first:** Facilitate them telling their parents while we listen in or facilitate the conversation for them (when teen is being seen in person).
  - **FOR ALL:** Get them <u>immediate</u> help, via:
    - Calling them an ambulance (911) and stay on the phone with them while it comes if they are with you at UIC, you can walk them to the ER

      OR
    - Ask if they feel comfortable taking themselves (in all instances, ensure the participant is not alone); stay on the phone with them.

        - If this needs to be facilitated, use a script like the following:
            - Does anyone close to you know you've been having these thoughts? Who is that person? Are they with you right now? OR My job is to make sure you/your child stay/s safe and it sounds like these thoughts may make this hard right now. Is there someone you can share these feelings with? Maybe a relative or friend?
- Consult with Dr. Jacobs immediately (O: 312-413-9178, all externs have 24 hour cell phone number). This can be done with or without the subject in the room, depending on the circumstances. Explain to the subject that you need to consult with your supervisor whenever someone expresses suicidal thoughts. If she is not available, please call the psychiatry resident on call at 312-996-7000.
- Follow up with the child's C2 and CHW via consensus notes. "Link" siblings if applicable.
- It is important to tell the subject that if they should ever have suicidal feelings in the future, they should tell someone about it, rather than keep these thoughts secret. Ask them if they think they would be able to do this. Ask them who they might talk to.
- Generally, any CHECK participant (parent or child) with current or past suicidal thoughts should be referred out and/or back to their PCP.

VIII. Child Abuse Protocol

- Assessors should always have a copy of the harm to others protocol questions with them whenever they are interviewing a subject. It is helpful to place the critical incident form in a separate manila folder that you bring with you to do interviews.

- If a subject discloses thoughts or actions of harm to others, or reports that someone is harming them, the interviewer immediately transitions to ask "harm to others" questions *("I'm going to ask you some more questions about this").* These questions should be asked when concerns are present or in the past. Abuse or harm to others can involve child abuse (physical, sexual, or neglect), homicidal ideation, or family/domestic violence (harm of any kind between siblings or others in home that goes beyond sibling/child rivalry to include physical violence, sexual abuse, or threats of harm).

- The safety monitoring staff on duty (the Principal Investigator, PI) will be the first point of contact when harm to others is encountered. The PI should always be consulted when harm to others is expressed; immediately or within 24 hours depending on the circumstances. If the PI is not available then the second point of contact, Rachel Jacobs, should be contacted.

- Questions regarding disclosed abuse (thoughts or actions):

        - Assessor will ask the following questions to clarify incident or thoughts disclosed:
            - Can you tell me who has hurt you/who you hurt/who you want to hurt?
            - Does [other party] live with you? How often do you see them?
            - Can you tell me what happened/what you have thought about doing? (probing questions can be used to ascertain type of harm, area of body where harm occurred, etc.)
            - When did this happen/when have you thought about doing this?
            - How often has this happened?
            - Who have you told?
            - Did anyone need any medical attention? Were there any bruises or marks?
            - Were the police called/involved?
            - Address and age of parties involved
            - Inform the person that we will need to report the incident, assess whether danger to the individual will increase as a result of the report. If so, determine whether it is necessary to call 911 or create a safety plan for the interim before DCFS can assess the situation.
        - Answers to above questions are needed for reporting purposes
    - Critical Incident Form:
        - Complete this form for any current or past harm that has occurred or if thoughts of harming others are reported
        - File this form …….
        - Prompt reporting to the Department of Children and Family Services (DCFS; Child Abuse Hotline at 800-25-ABUSE (800-252-2873 or TTY 1-800-358-5117).
        - Document DCFS Report in …..
    - What to do when Abuse or thoughts of abuse are disclosed:
        - It is important to tell the subject the following:
            - They should tell someone about it, rather than keep these actions or thoughts to themselves
            - Ask them if they think they would be able to do this
            - Ask them whom they might talk to
    - If subject is in immediate danger call 911 and stay with them until help arrives.
    - A referral to psychiatry should be offered if threat is not imminent
    - When child abuse is reported, a report will be made to DCFS by MHP staff within 24 hours of disclosure.
    - Per University of Illinois at Chicago Policy, the Campus Police will be notified that DCFS report has been made.
- Guidelines for Consulting MHP Supervisor (Rachel Jacobs)
    - Consult with the Supervisor immediately if:

- There is ongoing abuse or concern for the immediate safety of others
- If severity of harm cannot be ascertained from participant, MHP staff will contact local police to conduct a wellness check.
- Consult with the MHP Supervisor within 24 hours if incident is past and no current threat of harm to any individual involved
- Follow-up
    - Disclosure to family that a report will be made is recommended but not necessary. Discussion with the family of any report is to be done by a MHP staff (MHP Supervisor can be present if needed).
    - If it is determined that family notification is needed to keep someone safe, confidentiality will be broken as outlined the in the UI Health Privacy Policy ("We may disclose your health information to a public health authority that is authorized by law to receive reports of abuse or neglect, including reporting child and sexual abuse. In addition, we may disclose your health information if we believe you may be a victim of abuse, neglect, or domestic violence to the governmental agency or entity authorized to receive such information. This disclosure will be made consistent with the requirements of applicable federal and state laws").
    - MHP staff will follow-up with family in one week to ensure family has a plan regarding follow-up care. If no connection has been made, MHP staff will follow up with CHW and Care Coordinator to determine next steps appropriate to ensure safety of family. Documentation in Consensus outlining all steps taken.
