# Supplementary material for: Effect of Comprehensive Care Coordination on Medicaid Expenditures Compared With Usual Care Among Children and Youth With Chronic Disease: A Randomized Clinical Trial
Source: JAMA Netw Open. 2019 Oct 4;2(10):e1912604. doi: 10.1001/jamanetworkopen.2019.12604 (PMC6784784; doi:10.1001/jamanetworkopen.2019.12604)
Supplement: Supplement 2. — eTable 1. Codes Used to Identify Specific Chronic Diseases for CHECK Eligibility eTable 2. Demographic Information of Outliers eTable 3. Mean Expenditures, Before and After Intervention, of Outliers eFigure. Mean Expenditures, Before and After Intervention, by 6-Month Increments, Showing Parallel Trends [file jamanetwopen-2-e1912604-s002.pdf]

Supplementary Online Content

Caskey R, Moran K, Touchette D, et al. Effect of comprehensive care coordination on Medicaid expenditures compared with usual care among children and youth with chronic disease: a randomized clinical trial. *JAMA Netw Open*. 2019;2(10):e1912604. doi:10.1001/jamanetworkopen.2019.12604

eTable 1. Codes Used to Identify Specific Chronic Diseases for CHECK Eligibility

eTable 2. Demographic Information of Outliers

eTable 3. Mean Expenditures, Before and After Intervention, of Outliers

eFigure. Mean Expenditures, Before and After Intervention, by 6-Month Increments, Showing Parallel Trends

This supplementary material has been provided by the authors to give readers additional information about their work.

© 2019 Caskey R et al. *JAMA Network Open.*

eTable 1. Codes Used to Identify Specific Chronic Diseases for CHECK Eligibility

Diagnosis	ICD-9 code	ICD-10 code
**Asthma**	493.xx	J44.x, J45.xxx
**Diabetes**	250.xx	E10.xxxx, E11.xxxx, E13.xxxx
**Epilepsy**	345.xx	G40.xxx
**Prematurity**	765.00, 765.01, 765.02, 765.03, 765.04, 765.05, 765.06, 765.10, 765.11, 765.12, 765.13, 765.14, 765.15, 765.16, 765.20, 765.21, 765.22, 765.23, 765.24, 765.25, 765.26<br><br>768.5x, 768.6x, 768.7x, 768.9x	P07.0x, P07.1x, P07.2x, P07.30, P07.31, P07.32, P07.33, P07.34, P07.35<br><br>P84, P91.6x
**Sickle cell disease**<br><br>**Required at least a 3:1 ratio of the number of claims with ICD code for sickle cell disease vs. sickle cell disease trait*	282.41, 282.42, 282.6x, 289.52, 517.3x<br><br>Sickle cell disease trait:<br>282.5x	D57.0x, D57.1, D57.2xx, D57.4xx, D57.8xx<br><br>Sickle cell disease trait:<br>D57.3

© 2019 Caskey R et al. *JAMA Network Open.*

eTable 2. Demographic Information of Outliers

	Enrolled (n = 5)		Usual Care (n = 9)	
	n (%)		n (%)	
**Age at enrollment, *years***				
Mean (SD)	7.2	(10.7)	6.1	(9.5)
Median (IQR)	0.0	(12.0)	0.0	(13.0)
Adults (≥18)	1	(20.0)	2	(22.2)
Adolescents (13-17)	0	(0.0)	1	(11.1)
Children (6-12)	1	(20.0)	0	(0.0)
Young Children (1-5)	0	(0.0)	0	(0.0)
Infants (<1) [a]	3	(60.0)	6	(66.7)
**Gender**				
Male	3	(60.0)	5	(55.6)
Female	2	(40.0)	4	(44.4)
**CHECK diagnoses**				
Asthma	3	(60.0)	5	(55.6)
Diabetes	0	(0.0)	0	(0.0)
Prematurity	3	(60.0)	6	(66.7)
Seizure disorder	2	(40.0)	1	(11.1)
Sickle cell disease	0	(0.0)	0	(0.0)
No CHECK diagnosis	*0*	*(0.0)*	*0*	*(0.0)*
**Baseline risk tier**				
Low	0	(0.0)	2	(22.2)
Medium	3	(60.0)	4	(44.4)
High	2	(40.0)	3	(33.3)
**CDPS risk score**				
Mean (SD)	3.0	(2.1)	3.2	(2.2)
Median (IQR)	2.9	(2.0)	2.8	(3.0)

SD standard deviation; *IQR* interquartile range; *CDPS* Chronic Illness and Disability Payment System

[a] Excluded from program cohort due to lack of 12-month baseline period

© 2019 Caskey R et al. *JAMA Network Open.*

eTable 3. Mean Expenditures, Before and After Intervention, of Outliers

	Enrolled (n = 5)		Usual Care (n = 9)	
	mean (SD)		mean (SD)	
**Mean Expenditures**				
12-month pre	$150,119	($93,588)	$238,767	($238,083)
12-month post	$23,811	($50,303)	$46,988	($64,767)
**Inpatient Cost, *$***				
12-month pre	$144,539	($96,580)	$211,677	($235,655)
12-month post	$12,993	($29,053)	$30,179	($59,388)
**ED Cost, *$***				
12-month pre	$342	($587)	$423	($752)
12-month post	$955	($1,647)	$384	($655)
**Outpatient Cost, *$***				
12-month pre	$622	($462)	$508	($520)
12-month post	$506	($528)	$442	($727)
**RX Cost, *$***				
12-month pre	$2,861	($4,202)	$6,225	($4,913)
12-month post	$2,966	($6,600)	$2,277	($3,932)
**Other Cost, *$***				
12-month pre	$1,756	($1,447)	$19,933	($37,857)
12-month post	$6,391	($12,559)	$13,705	($28,537)

© 2019 Caskey R et al. *JAMA Network Open.*

eFigure. Mean Expenditures, Before and After Intervention, by 6-Month Increments, Showing Parallel Trends

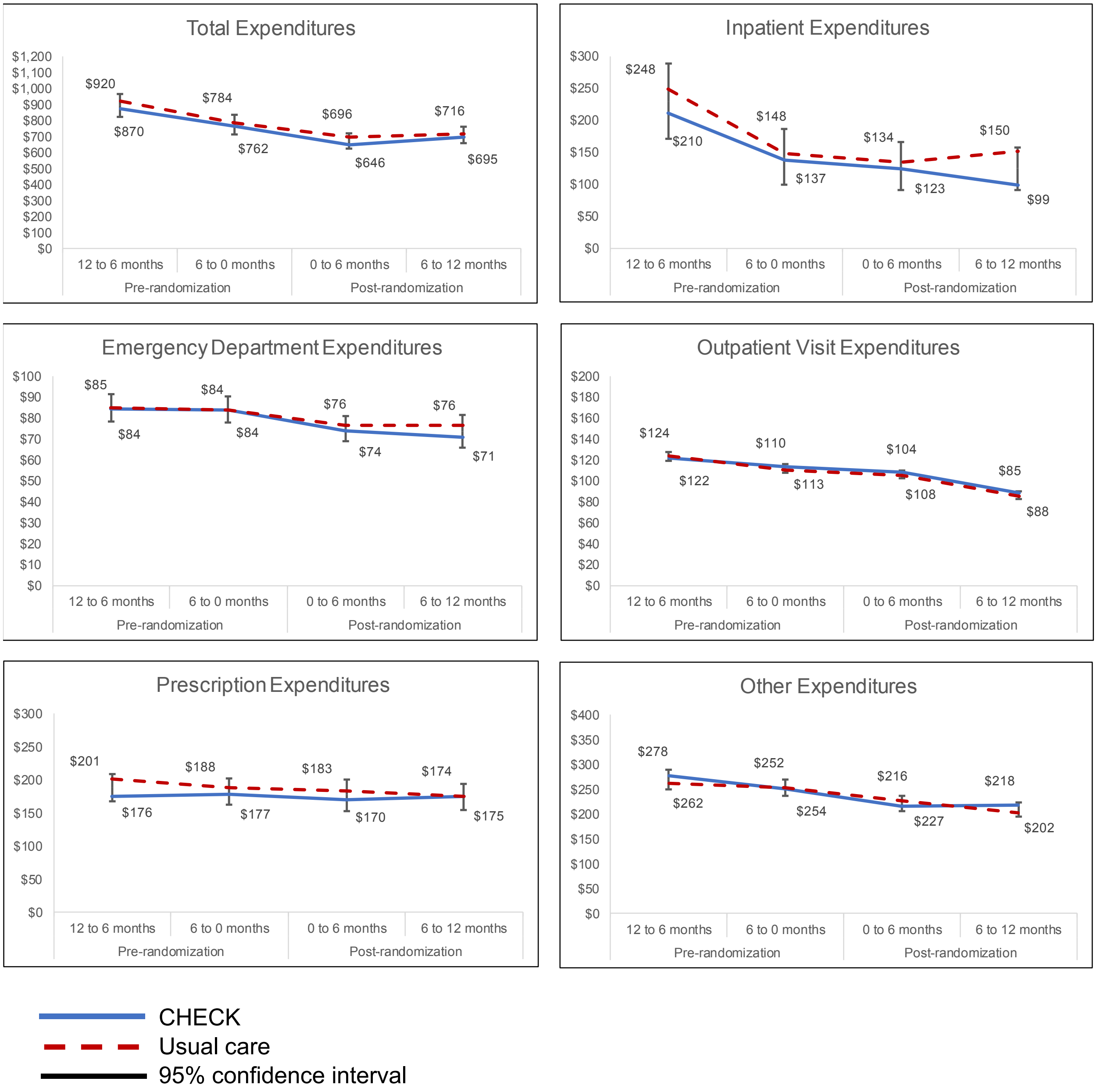

© 2019 Caskey R et al. *JAMA Network Open.*
