# Supplementary material for: Effect of Comprehensive Care Coordination on Medicaid Expenditures Compared With Usual Care Among Children and Youth With Chronic Disease: A Randomized Clinical Trial
Source: JAMA Netw Open. 2019 Oct 4;2(10):e1912604. doi: 10.1001/jamanetworkopen.2019.12604 (PMC6784784; doi:10.1001/jamanetworkopen.2019.12604)
Supplement: Supplement 3. — Data Sharing Statement [file jamanetwopen-2-e1912604-s003.pdf]

Data Sharing Statement

Caskey. Effect of Comprehensive Care Coordination on Medicaid Expenditures Compared With Usual Care Among Children and Youth With Chronic Disease. *JAMA Netw Open*. Published October 04, 2019.
10.1001/jamanetworkopen.2019.12604

Data
Data available: No

Additional Information
Explanation for why data not available: Illinois Healthcare and Family Services does not permit sharing of Medicaid claims data.
